# Murine obscurin and Obsl1 have functionally redundant roles in sarcolemmal integrity, sarcoplasmic reticulum organization, and muscle metabolism

**DOI:** 10.1038/s42003-019-0405-7

**Published:** 2019-05-09

**Authors:** Jordan Blondelle, Valeria Marrocco, Madison Clark, Patrick Desmond, Stephanie Myers, Jim Nguyen, Matthew Wright, Shannon Bremner, Enrico Pierantozzi, Samuel Ward, Eric Estève, Vincenzo Sorrentino, Majid Ghassemian, Stephan Lange

**Affiliations:** 1 Division of Cardiology, School of Medicine, University of California, San Diego, 92093 CA USA; 2 Department of Orthopedic Surgery, School of Medicine, University of California, San Diego, 92093 CA USA; 3 Molecular Medicine Section, Department of Molecular and Developmental Medicine, University of Siena, Siena, 53100 Italy; 4 Université Grenoble Alpes, HP2, Grenoble, 38706 France; 5 Department of Chemistry and Biochemistry, University of California, San Diego, 92093 CA USA; 6 Wallenberg Laboratory, Department of Molecular and Clinical Medicine, Institute of Medicine, University of Gothenburg, Gothenburg, 413 45 Sweden

**Keywords:** Mechanisms of disease, Membrane proteins, Molecular medicine, Experimental models of disease

## Abstract

Biological roles of obscurin and its close homolog Obsl1 (obscurin-like 1) have been enigmatic. While obscurin is highly expressed in striated muscles, Obsl1 is found ubiquitously. Accordingly, obscurin mutations have been linked to myopathies, whereas mutations in Obsl1 result in 3M-growth syndrome. To further study unique and redundant functions of these closely related proteins, we generated and characterized Obsl1 knockouts. Global Obsl1 knockouts are embryonically lethal. In contrast, skeletal muscle-specific Obsl1 knockouts show a benign phenotype similar to obscurin knockouts. Only deletion of both proteins and removal of their functional redundancy revealed their roles for sarcolemmal stability and sarcoplasmic reticulum organization. To gain unbiased insights into changes to the muscle proteome, we analyzed tibialis anterior and soleus muscles by mass spectrometry, uncovering additional changes to the muscle metabolism. Our analyses suggest that all obscurin protein family members play functions for muscle membrane systems.

## Abstract (web summary)

Blondelle & Marrocco et al. show that deletion of both Obsl1 and obscurin in skeletal muscle leads to severe alterations in sarcolemma organization, protein expression profiles, and cellular calcium handling. These alterations affected muscle physiology and suggest redundant roles for Obsl1 and obscurin in murine muscle function.

## Introduction

Giant muscle proteins have been known for a long time to play important functions for skeletal and cardiac development and function, as well as for pathology of myopathies. Among the best characterized of these cellular giants are titin (also called connectin; approx. 3.5 MDa), and obscurin (approx. 800 kDa). Similar to titin, proteins of the obscurin protein family combine structural with signaling functions. Obscurin, the biggest mammalian member of this protein family, consists of serially arranged immunoglobulin-like and fibronectin-type III domains that are interspersed with signaling domains^1,2^. Unlike titin, obscurin contains in addition to its protein kinase domains also a calcium/calmodulin-binding IQ motif, as well as a RhoGEF domain triplet (SH3-DH-PH domains). Extensive splicing of the obscurin gene results in at least three main transcripts, the giant obscurin-A and obscurin-B splice isoforms, and the obscurin kinase only (also known as KIAA1639 or Obsc-kin), which originates from a separate promoter^3^. Recent reports indicate the presence of smaller obscurin splice variants^4^, their expression in non-muscle tissues, and important functions for tumorigenicity and metastasis^5–7^. Other members of the obscurin protein family are the ubiquitously expressed obscurin-like 1 (Obsl1)^8^ as well as the striated muscle/atrial preferentially expressed protein kinase (Speg, also known as Apeg)^9^. Evolutionary, all members of the obscurin family are thought to have originated from one ancestral gene^2,8^. This idea is supported by the fact that invertebrates like *Caenorhabditis elegans* have one obscurin family ortholog called unc-89^10^. In addition, Obsl1 and Speg show sequence similarity to the obscurin N and C terminus, respectively, and at least for Obsl1 also a certain degree of functional redundancy^8,11^.

Knockout models for obscurin and Speg have been helpful to delineate biological functions of these genes/proteins. While the knockout for obscurin resulted in a mild skeletal myopathy, changes to the sarcoplasmic reticulum (SR) and membrane fragility after exercise^12–14^, Speg knockouts displayed a prominent dilated cardiomyopathy, disruption of the junctional SR membrane, and centronucleolar myopathy^15–17^.

Lately, it emerged that mutations in the human Obsl1 result in 3M-growth syndrome in affected patients. On the molecular level, many of the human Obsl1 mutations are thought to result in nonsense-mediated decay of its messenger RNA (mRNA) and ultimately loss of the protein. However, owing to the extensive splicing displayed by Obsl1^8^ (Supplementary Fig. 1a), detailed investigations into which isoforms are affected/unaffected and their respective expression levels in patient tissues remain to be done.

The sarcomeric proteins titin and myomesin-1 have been identified as interaction partners for both obscurin and Obsl1. Titin offers two binding sites to obscurin: the titin C-terminal Ig-domain M10 interacts with obscurin Ig-domain 1^11^, while titin domains Z9–Z10 were identified to bind to obscurin Ig domains Ig48-Ig49 (also called Ig58-Ig59, depending on the obscurin splice isoform)^2^. Interaction of obscurin with the titin C terminus is the predominant binding site in mature myofilaments, giving rise to the prominent M-band colocalization of obscurin. The titin binding site in Ig-domain 1 of obscurin is evolutionary conserved for Obsl1 Ig-domain 1, albeit with a higher affinity compared to obscurin^18^. Differences in the side chains in obscurin vs. Obsl1 that generate the titin interaction interface and account for the differential binding affinity also contribute to the slightly different intracellular sorting of obscurin vs. Obsl1. Mutations in titin Ig-domain M10 that are known to cause limb-girdle muscular dystrophy 2J in affected patients were shown to disrupt the interaction with obscurin or Obsl1^11^. Indeed, biochemical analyses of the various titin mutations found in titin Ig-domain M10 indicated that the severity of the muscular dystrophy correlates with the degree of loss of interaction to obscurin or Obsl1.

The functional redundancy between Obsl1 and obscurin can also be seen in their association with myomesin-1^11^. Recent advances in the co-crystallization of this interaction revealed that binding of myomesin-1 to obscurin or Obsl1 Ig3 is necessary for proper folding of their Ig domains in a hitherto unprecedented trans-complementation mechanism^19^.

Another well-characterized binding site for a muscle-specific isoform of ankyrin-1 (sAnk1.5) is located within the obscurin-A isoform C terminus^20,21^. Complex formation between obscurin, sAnk1.5, and tropomodulin-3 was demonstrated to be important for SR architecture and function^12,22,23^, and stability of sAnk1.5 itself. Indeed, we demonstrated that loss of obscurin leads to increased sAnk1.5 turnover in a cullin-3/KCTD6-dependent manner^24^. Intriguingly, the functional property that obscurins may regulate the stability and turnover of their interaction partners may be conserved within this protein family, as well as evolutionary: Obsl1 interacts with cullin-7^25,26^, and dysfunction of this E3-ligase complex by mutations in cullin-7 or Obsl1 have been linked to the development of 3M-growth syndrome in patients^27,28^. Moreover, the invertebrate obscurin homolog unc-89 directly interacts with cullin-1, and regulates myosin filament organization in a MEL-26/cullin-3- and MEI-1 (katanin)-dependent way^24,29^.

In this study, we set out to further investigate biological functions for obscurin proteins for skeletal muscles, with a special emphasis to uncover functional redundancies between obscurin and Obsl1. As Obsl1 is ubiquitously expressed and its mutations lead to a growth disorder in affected patients, we also investigated the phenotype of global Obsl1-knockout mice. Our studies uncovered that global Obsl1 knockouts are embryonically lethal, while skeletal muscle-specific Obsl1 knockouts show a mild phenotype similar to obscurin knockouts. Only deletion of both proteins in skeletal muscles uncovered their role for SR, cellular calcium storage and handling, dystrophin–sarcoglycan (DSG) complex, and sarcolemmal integrity, as well as changes to the muscle metabolism.

## Results

### Global knockout mice for Obsl1 are embryonically lethal

We generated the Obsl1 conditional knockout mouse to test the effect that ablation of Obsl1 would have on the whole organism, and on skeletal muscle organization and function. Due to its extensive splicing^8^ and the presence of alternative start codons, we decided to place coding exons 1 through 4 of the murine *Obsl1* locus within LoxP sites (Supplementary Fig. 1a). This strategy should result in the total ablation of any Obsl1 isoform (Supplementary Fig. 1b). By crossing gene-targeted mice, with protamine-Cre recombinase mice^30^, we generated global *Obsl1* knockouts. Surprisingly, global ablation of *Obsl1* in mice results in embryonic lethality before embryonic day E8 (Table 1), while mice heterozygous for *Obsl1* developed normally. These results are in contrast to reports on human patients carrying various mutations in *Obsl1*, which are thought to result in nonsense-mediated decay of its mRNA in a majority of cases. Clinically, these patients present with the development of 3M-growth syndrome^28,31^.

**Table 1:** Genotype analysis of global Obsl1-knockout mice

Genotype	Number and % of embryos/animals in genotyping analysis				Expected Mendelian ratios
	E7–E8.5	E10.5–E13.5	E17–E18	P21	
Control (+/+)	7 (30%)	15 (44%)	4 (33%)	37 (36%)	25%
Heterozygous (+/−)	16 (70%)	19 (56%)	8 (67%)	66 (64%)	50%
Knockout (−/−)	0 (0%)	0 (0%)	0 (0%)	0 (0%)	25%

*Obsl1* obscurin-like 1

### Obsl1 and/or obscurin are not required for sarcomerogenesis and skeletal muscle development

**Fig. 1:**
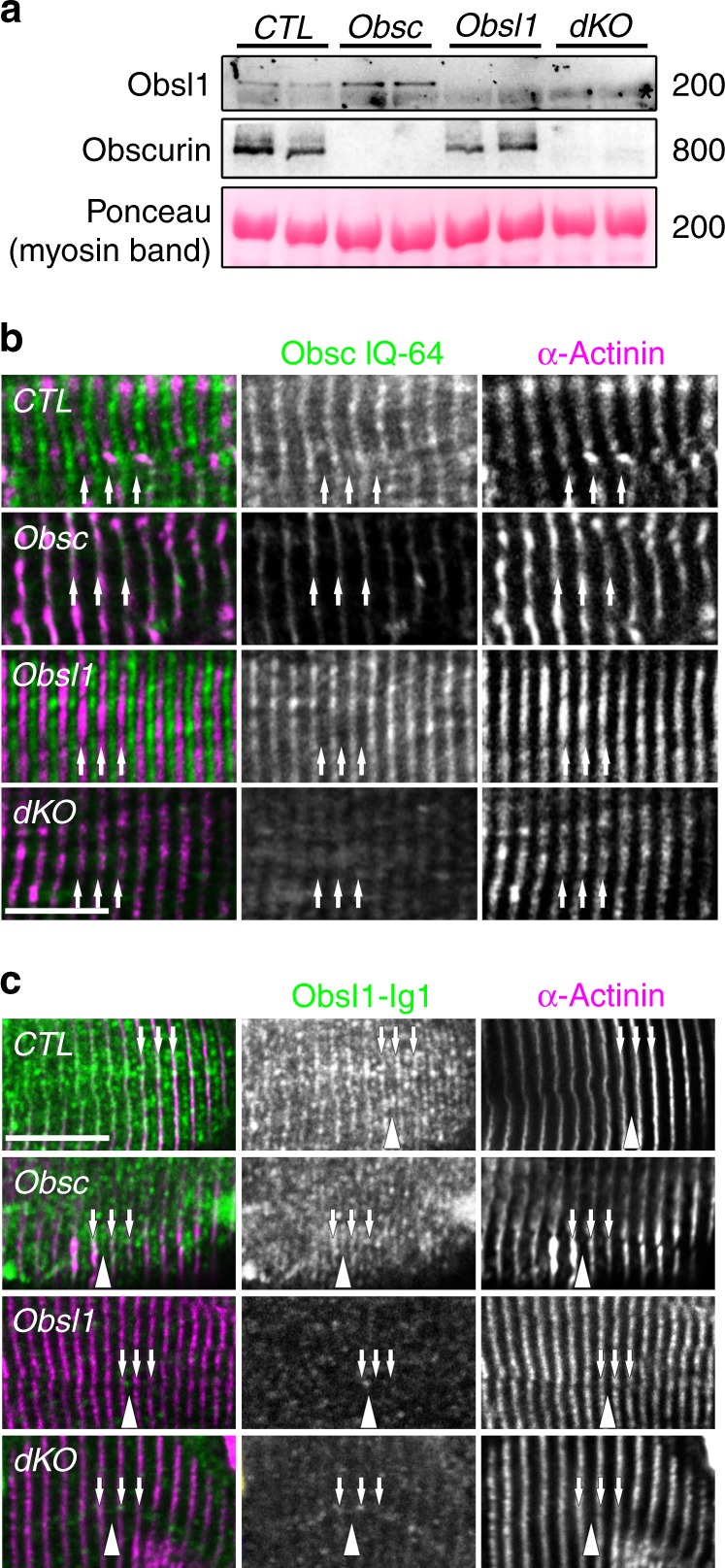
Expression and localization of obscurin and Obsl1 (obscurin-like 1) in tibialis anterior (TA) muscles. **a** Expression levels of obscurin and Obsl1 in TA muscles from obscurin-knockout (Obsc), Obsl1 skeletal muscle-knockout (Obsl1), and double-knockout (dKO) mice, compared to controls (CTL). Asterisk denotes non-specific cross-reactivity of the secondary antibody with myosin. Myosin band in Ponceau stain as a loading control. **b**, **c** Sarcomeric localization of obscurin (**b**, IQ-64 epitope antibody) or Obsl1 (**c**, Ig1 epitope antibody) in Obsc, Obsl1, and dKO, as well as control (CTL) TA muscles. Arrows indicate Z-disc localizations as indicated by α-actinin 2 counterstain, while arrowheads signify the sarcomeric M-band. Please note that some background staining caused by non-specific cross-reactivity of the antibodies is observed in knockout tissues. Scale bars = 10 µm

To overcome the lethality, we crossed conditional *Obsl1* mice with mice expressing Cre recombinase under control of the myogenin promoter^32^. These mice do not develop gross morphological abnormalities or display premature death. However, it was shown that obscurin and Obsl1 display functional redundancy for two of their common interaction partners, namely myomesin-1 (Myom1) and titin’s C-terminal domain M10^11^. Loss of obscurin was shown to result in a mild myopathy phenotype, without adversely affecting the animals at baseline^12^. To investigate if the lack of a more pronounced skeletal muscle phenotype might be due to a rescue by Obsl1, we generated skeletal muscle-specific double knockouts (dKO; Fig. 1a). dKO mice were viable, fertile, and developed normally. Deletion of *obscurin* led to slightly increased protein levels in Obsl1 (normalized protein levels 2.5 ± 0.1 in obscurin vs. 1.0 ± 0.02 in CTL; *p* < 0.001, *n* = 4), while obscurin expression was unchanged in *Obsl1*-knockout TA muscles (Fig. 1a, Supplementary Fig. 1c). Microscopic analysis of myofilament structure using sarcomeric α-actinin 2 indicated normal sarcomerogenesis (Fig. 1b, c, Supplementary Fig. 1d, h). Loss of *Obsl1* or *obscurin* (*Obsc*) does not alter localization of the other obscurin protein family member in tibialis anterior (TA) muscles (Fig. 1b, c). However, we noticed that two knockout validated Obsl1 antibodies recognizing different epitopes within the protein displayed differential localizations of Obsl1 within the sarcomere. An antibody that was raised against N-terminal Ig-domain 1 of the protein^11^ displayed M-band and Z-disc localization (Fig. 1c), while a commercially available antibody that recognizes Ig-domain 14 of Obsl1 localized almost exclusively to Z-discs (Supplementary Fig. 1d).

Loss of unc-89 in *Caenorhabditis elegans* and in *Drosophila* results in the disruption of myosin lattice organization, partially due to loss of the RhoGEF activity in the invertebrate obscurin homolog^33,34^. While searching for novel interaction partners for Obsl1 by yeast two-hybrid screening, we identified the C terminus of myosin (MyH6), dystonin (Dst), and filamin-C (FlnC) as putative binding partners (Supplementary Fig. 1a, lower panel). Both dystonin and FlnC were previously identified in another high-throughput affinity assay as putative Obsl1 interaction partners^35^. Analysis of myosin binding to Obsl1 by co-immunoprecipitation validated their association (Supplementary Fig. 1e). However, looking at protein expression and localization for some of the newly and previously reported putative interaction partners like myomesin-1, no overt changes in protein levels, organization, and subcellular localization were observable in immunoblots or immunofluorescence images of longitudinal TA sections (Supplementary Fig. 1f, g). In addition, unlike in *C. elegans* or *Drosophila*, loss of obscurin and/or Obsl1 did not result in markedly altered Z-disc and M-band structures (Supplementary Fig. 1h).

**Fig. 2:**
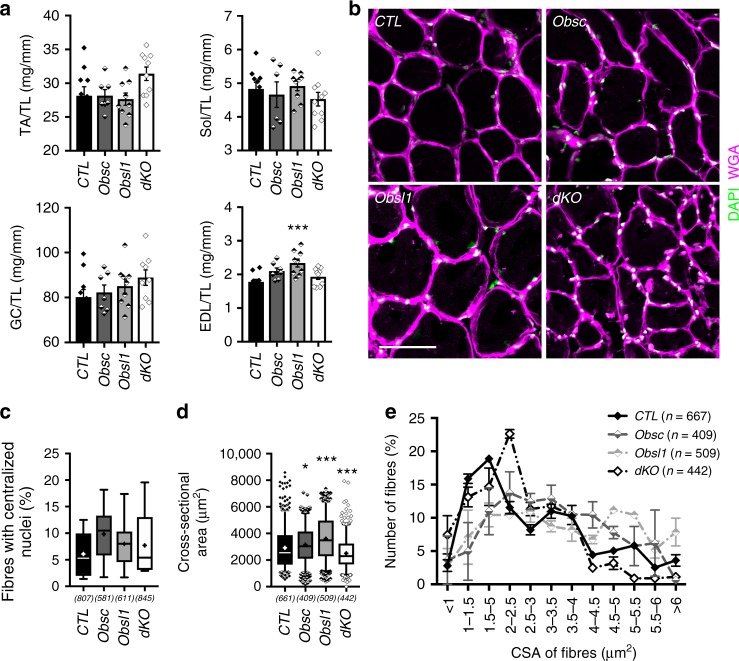
Loss of obscurin and/or Obsl1 (obscurin-like 1) does not influence muscle weights, and has little impact on cross-sectional area and muscle regeneration. **a** Muscle weight to tibia-length ratios of tibialis anterior (TA), soleus (Sol), gastrocnemius (GC), and extensor digitorum longus (EDL) muscles in control (CTL), obscurin knockout (Obsc), Obsl1 skeletal muscle-knockout (Obsl1), and double-knockout mice (dKO). Sample sizes (*n*) are indicated in the figure; ***p < 0.001 vs. CTL. **b**–**e** Wheat germ agglutinin (WGA)- (magenta) and 4′,6-diamidino-2-phenylindole (DAPI)- (green) stained immunofluorescence of frozen TA cross-sections (**b**) used to determine fibers with centralized nuclei (**c**) and cross-sectional fiber areas for each group (**d**, **e**). Scale bar = 200 µm. Muscle from three mice per group were analyzed, counted fibers (*n*) and *p* values are indicated in figure (**c**–**e**). *p < 0.05, ***p < 0.001 vs. CTL. Box and whisker plots in **c** and **d** depict 5–95 percentile, averages (as cross) and outliers

We next wondered what impact the loss of Obsl1 and/or obscurin would have on muscle mass and cross-sectional muscle area. Normalized muscle masses for TA, soleus (Sol) and gastrocnemius (Gas), was unchanged in 4-month-old animals. We observed only a small increase in the mass of extensor digitorum longus (EDL) muscle of Obsl1-knockout animals (Fig. 2a). No significant changes were observed in the number of centralized nuclei in TA muscle at 4 months of age (Fig. 2b, c), confirming earlier observations for muscles from obscurin knockouts^12^. Study of cross-sectional areas in sections of TA muscle revealed increases in fiber sizes in Obsl1 and obscurin-knockout mice, probably responsible for the slight increase in muscle mass of the Obsl1 animals, while dKO mice displayed decreases in fiber sizes, specifically of fibers with high cross-sectional areas (Fig. 2d, e).

### Loss of obscurin/Obsl1 impacts the DSG complex and its associated proteins

**Fig. 3:**
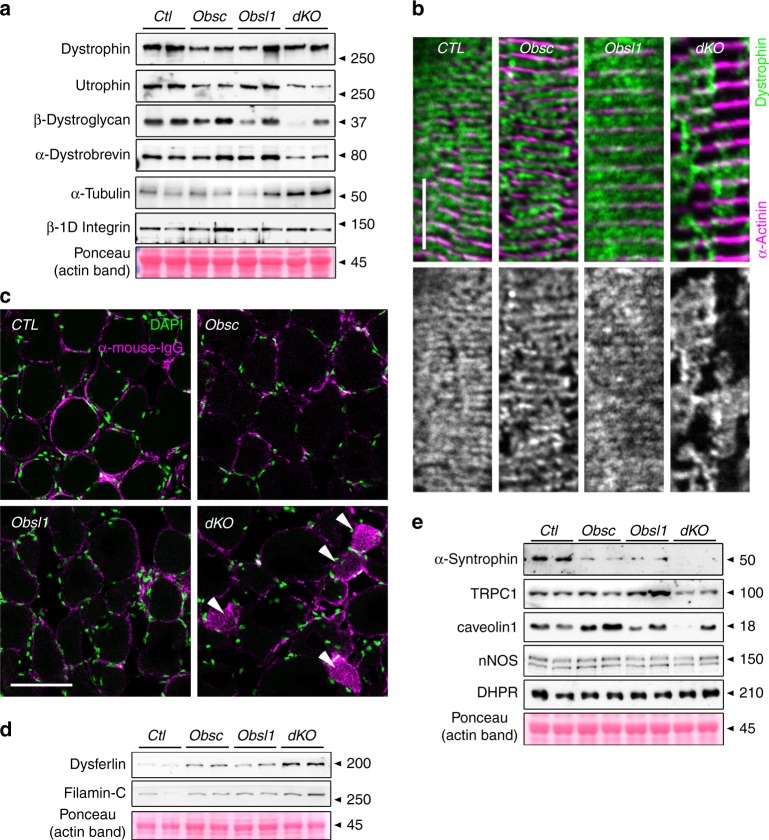
Loss of obscurin and Obsl1 (obscurin-like 1) impacts dystrophin–sarcoglycan (DSG) complex and its associated proteins, membrane integrity and membrane repair mechanisms. **a** Expression levels of DSG complex proteins dystrophin, utrophin, β-dystroglycan, and α-dystrobrevin, as well as α-tubulin and β1D-integrin in whole protein lysates of tibialis anterior (TA) muscles of control (CTL), obscurin-knockout (Obsc), Obsl1 skeletal muscle-knockout (Obsl1), and double-knockout (dKO) mice. Ponceau-stained actin band is shown as a loading control. **b** Subsarcolemmal dystrophin localization in longitudinal sections of CTL, Obsc, Obsl1, and dKO TA muscles. Sarcomeric α-actinin 2 was used as counterstain. Scale bar = 10 µm. **c** Mouse immunoglobulin (magenta) and 4′,6-diamidino-2-phenylindole (DAPI) stain (green) of frozen cross-sections of CTL, Obsc, Obsl1, and dKO TA muscles. Arrowheads indicate immunoglobulin G (IgG)-positive muscle fibers. Scale bar = 200 µm. **d**, **e** Protein expression levels of dysferlin and Filamin-C (**d**), as well as DSG-associated proteins α-syntrophin, Trpc1, caveolin-1, neuronal nitric oxidase (nNOS), and l-type calcium channel DHPR α-2 subunit (**e**) in whole lysates of CTL, Obsc, Obsl1, and dKO TA muscles. Ponceau-stained actin is shown as a loading control

Obscurin has been reported to be important for sarcolemmal integrity, and loss of the protein results in changes to the subsarcolemmal microtubule network, increased membrane fragility, and reduced running performance of mice^13,14^. We investigated if changes to sarcolemmal integrity and the DSG complex and its associated proteins are exacerbated in Obsl1 and/or dKO mice. Loss of both obscurin and Obsl1 reduced protein levels of some DSG components, while levels of tubulin increased slightly (*p* = 0.06). In addition, overall levels of dystrophin and β1D- integrin remained unchanged (Fig. 3a, Supplementary Figs. 2a, 7a). However, analysis of subsarcolemmal dystrophin localization in longitudinal sections of TA muscles revealed a patchy and abnormal distribution of the protein in obscurin knockouts, but more so in dKO mice, indicating enhanced membrane fragility even at baseline (Fig. 3b, Supplementary Fig. 2b). Indeed, when investigated for the presence of mouse immunoglobulins in immunofluorescence assays, a significant portion of muscle fibers in dKO mice stained positive (Fig. 3c, Supplementary Fig. 2c). Increased membrane fragility in dKO muscles also leads to significantly enhanced expression of dysferlin (Dysf) and FlnC, proteins involved in repair and compensatory mechanisms (Fig. 3d, Supplementary Figs. 2d, 7a).

The DSG complex also regulates expression and function of sarcolemma-based membrane channels and signaling. Specifically, α-syntrophin has been demonstrated to establish a link between dystrophin/the DSG complex and sarcolemmal ion channels, such as Trpc1 as well as caveolae components and neuronal nitric oxidase (nNOS) signaling. When investigated, we noticed reduced caveolin-1 and Trpc1 levels in TA muscles of dKO mice, while nNOS remained unchanged (Fig. 3e, Supplementary Figs. 2e, 7a). Levels of L-type calcium channel remained also comparable with controls, indicating potential changes to DSG-associated, but not T-tubule-based ion channels.

### Proteome analysis of knockout muscles

**Fig. 4:**
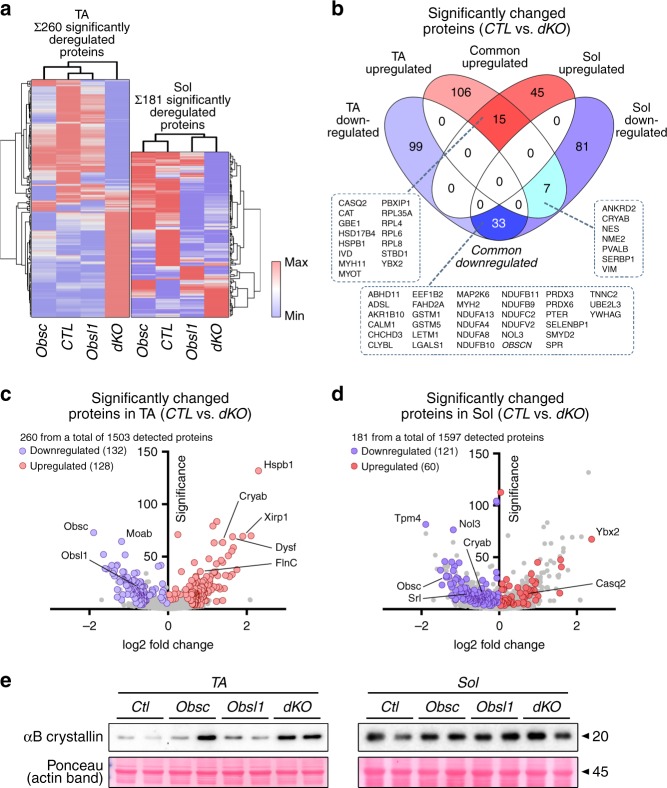
Proteome analysis of soleus (Sol) and tibialis anterior (TA) muscles. **a** Hierarchical clustering of significantly altered proteins identified in TA and Sol muscles of control (CTL), obscurin-knockout (Obsc), skeletal muscle-specific Obsl1 (obscurin-like 1)-knockout (Obsl1), and double-knockout (dKO) mice. **b** Grouping of significantly changed proteins in both muscles identified 15 proteins as common upregulated, while 33 proteins were found to be common downregulated between Sol and TA. The analysis also identified seven proteins that are differentially regulated between TA and Sol muscles. All commonly and differentially regulated proteins are identified in the boxes. **c**, **d** Volcano plot of significantly altered proteins between control (CTL) and dKO TA (**c**) and Sol (**d**) muscles. **e** Protein levels of αB-crystallin in whole TA and Sol muscle lysates from CTL, Obsc, Obsl1, and dKO mice. Ponceau-stained actin band is shown as a loading control

To reveal quantitative changes to the muscle proteome between control, obscurin-knockout, Obsl1-knockout, or dKO mice, we isolated proteins from TA and Sol muscles. We specifically focused on changes between control and dKO muscles to eliminate compensatory mechanisms between Obsl1 and obscurin that could potentially obfuscate results. Of the 1503 and 1597 detected proteins in TA and Sol of all genotypes, we identified 260 and 181 proteins that were significantly deregulated between dKO and control muscles, respectively (Fig. 4a, b, Supplementary Figs. 8, 9, Supplementary Data File 1). Gene ontology (GO) term and pathway analysis of significantly changed proteins from all investigated muscles, or Sol and TA alone revealed enrichment of oxidative phosphorylation, TCA (tricarboxylic acid) cycle, and electron transport chain, as well as muscle contraction processes (Supplementary Fig. 3a–c, Supplementary Data Files 2–4). Among the significantly upregulated proteins in TA muscles of dKO mice are FlnC and Dysf, corroborating results obtained in our immunoblot analyses (Figs. 3d, 4c, Supplementary Fig. 2d). Comparison of significantly changed proteins between TA and Sol revealed 15 proteins as commonly upregulated and 33 proteins as commonly downregulated in both analyzed muscle types, including calsequestrin-2 (Casq2), Hspb1 (also known as Hsp27), myotilin, or calmodulin-1 (Fig. 4b, Supplementary Data File 1). Intriguingly, we also noticed several proteins that were differentially regulated between Sol and TA, including αB-crystallin (Fig. 4c, d, Supplementary Figs. 2f, 7b). Indeed, when studied in immunoblot assays of protein lysates from TA and Sol muscles from all groups, only TA muscle of dKO mice showed significantly increased αB-crystallin levels, while αB-crystallin levels in Sol muscles remained largely unchanged (Fig. 4e, Supplementary Figs. 2f, 7b). These muscle type-specific differences may be reflective of the differential expression levels of obscurin splice isoforms observed in these muscles, and/or on the muscle type (fast vs. slow twitch)^12,36^.

### Removal of obscurin/Obsl1 alters SR protein levels

**Fig. 5:**
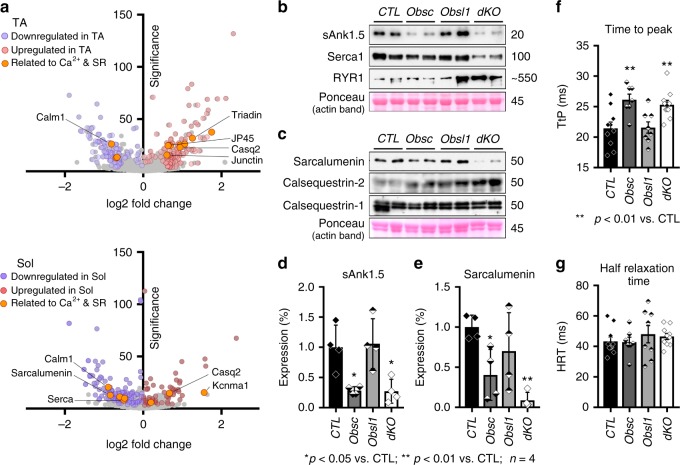
Changes to sarcoplasmic reticulum (SR) and associated proteins. **a** Volcano plots of significantly altered SR and its associated proteins identified in our proteome analysis of double-knockout (dKO) vs. control tibialis anterior (TA) and soleus (Sol) muscles. **b**–**e** Immunoblot analysis and quantification of SR-associated proteins in TA muscles, including small ankyrin-1.5 (sAnk1.5; **b**, **d**), sarcoendoplasmic reticulum Ca^2+^ ATPase (Serca1; **b**), and ryanodine receptor (RyR; **b**), as well as lumenal calcium-binding proteins sarcalumenin (**c**, **e**) and calsequestrin 1 and 2 (**c**). Ponceau-stained actin band is shown as a loading control (**b**, **c**). **d**–**g** Sample sizes (*n*) and *p* values are indicated in the figure. **f**, **g** Analysis of extensor digitorum longus muscle “time to peak” (**f**) and “half-relaxation time” (**g**). The full evaluation of twitch parameters and their statistical analyses are shown in Table 2

Our enrichment analysis revealed several proteins involved in muscle contraction, calcium handling, and signaling as significantly changed in dKO muscles. Among them, the SR proteins sarcoendoplasmic reticulum Ca^2+^ ATPase (Serca), sarcalumenin, triadin, JP-45 and junctin were found to be deregulated in dKO muscles (Fig. 5a). We corroborated suspected changes to the SR and its associated proteins by immunoblot analyses, and found a significant reduction of Serca levels, while ryanodine receptors (RyRs) were upregulated (Fig. 5b, Supplementary Figs. 2g, 7c). We also investigated levels of sAnk1.5, which was shown to be a binding partner for the obscurin-A C terminus. As shown before, loss of obscurin resulted in a decrease in sAnk1.5 levels due to increased turnover of the protein. Loss of Obsl1 did not affect sAnk1.5 levels, while muscles from dKO mice displayed sAnk1.5 levels comparable with those found in obscurin knockouts (Fig. 5b, d). Our proteome data also indicated changes to calcium-binding proteins located within the lumen of the SR. We tested protein levels of Casq1, Casq2, and sarcalumenin by immunoblot analyses of whole muscle lysates. Sarcalumenin levels decreased significantly between control and dKO muscles, while Casq2 protein levels increased, and Casq1 remained unchanged (Fig. 5c, e, Supplementary Figs. 2g, 7c).

These data indicate changes to cellular calcium handling in dKO muscles, which may be reflected in defective excitation contraction coupling and muscle contraction. To investigate if these molecular changes alter muscle physiology, we studied EDL twitch parameters in all groups (Table 2). EDL muscles of obscurin-knockout and dKO mice displayed increased time-to-peak (TtP) values compared to muscles from control and Obsl1 knockouts (Fig. 5f). However, half-relaxation times remained unchanged (Fig. 5g).

**Table 2:** Twitch parameter analysis on 4-month-old mice

Parameters	CTL	Obsc	Obsl1	dKO
Number of analyzed muscles (females/males)	10 (6/4)	7 (3/4)	9 (4/5)	10 (4/6)
TtP (ms)	21.45 ± 1.02	26.14 ± 0.91**	21.56 ± 0.92	25.3 ± 0.74**
HRT (ms)	43.3 ± 2.81	42.86 ± 3.20	48.00 ± 5.68	46.5 ± 1.99
Full-width at half-maximum (ms)	57 ± 3.4	60 ± 3.9	63 ± 6.3	64 ± 2.6
Stress during tetanus (kPa)	71 ± 3.8	99 ± 5**	79 ± 6.2	70 ± 5.8
PCSA (mm^2^)	0.51 ± 0.027	0.61 ± 0.023	0.6 ± 0.034	0.55 ± 0.03
EDL muscle mass (mg)	3.4 ± 0.19	4 ± 0.22	4.3 ± 0.28**	3.9 ± 0.18
Fiber length (mm)	6.2 ± 0.11	6.1 ± 0.16	6.7 ± 0.13*	6.5 ± 0.2

Values are presented as averages and standard error of mean. Samples sizes are shown in the table; *p* values are **p  < 0.01 or *p <   0.05 vs. CTL

*TtP* time to peak, *HRT* half-relaxation time, *PCSA* physiological cross-sectional area, *CTL* control, *Obsl1* obscurin-like 1, *dKO* double knockout, *EDL* extensor digitorum longus

### Metabolic changes in obscurin/Obsl1-knockout mice

**Fig. 6:**
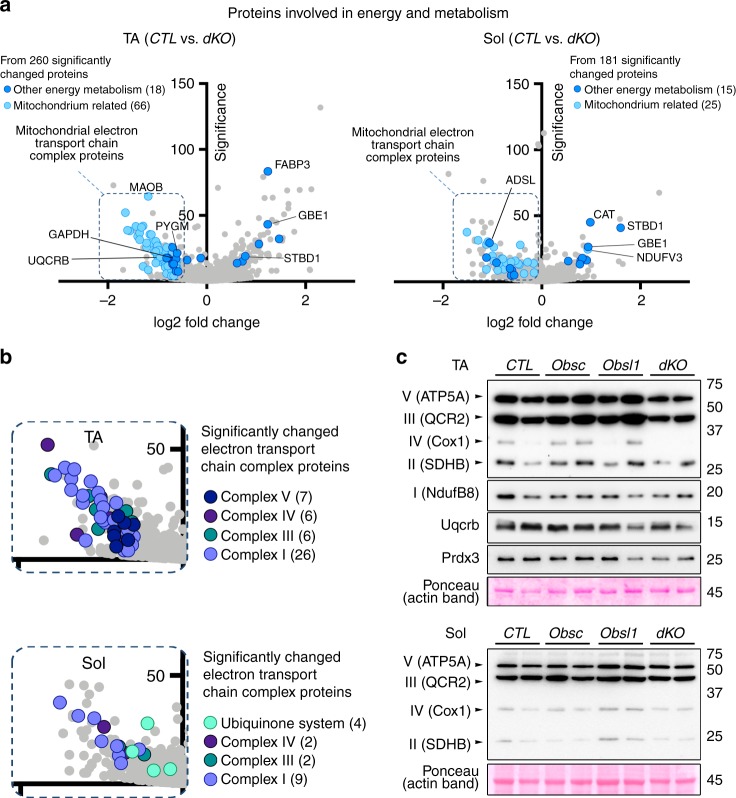
Analysis of changes to the muscle metabolism and mitochondria. **a** Volcano plot of significantly altered proteins between control (CTL) and double-knockout (dKO) tibialis anterior (TA; left panel) and soleus muscles (Sol; right panel) involved in energy metabolism (blue) and mitochondria (light blue). **b** Volcano plot of electron transport chain proteins identified in our proteome analysis as significantly altered between CTL and dKO TA and Sol muscles. The number of significantly changed proteins is indicated in brackets. **c** Analysis of mitochondrial electron transport chain complex protein levels as well as mitochondrially located peroxiredoxin-3 (Prdx3) in TA and Sol muscles from CTL, obscurin-knockout (Obsc), skeletal muscle-specific Obsl1 (obscurin-like 1)-knockout (Obsl1), and dKO mice. Ponceau-stained total actin band is shown as a loading control

A surprising result of the enrichment analysis was the large number of significantly changed proteins belonging to metabolic pathways and the mitochondrial electron transport chain (Fig. 6a). Specifically, we found increased levels of Gbe1 (1,4-α-glucan branching enzyme 1) and Stbd1 (starch binding domain 1) in dKO Sol and TA muscles. Both proteins are involved in glycogen metabolism, increasing solubility of glycogen by branching, and its breakdown through glycophagy, respectively. Conversely, the rate determining enzyme responsible for glycogen breakdown, muscle glycogen phosphorylase, was significantly decreased in dKO mice. We also found significantly decreased levels of monoamine oxidase A and B (Maoa, Maob) in TA muscles of dKO mice, while Sol muscle of dKO mice had increased levels of catalase, perhaps indicative of increased oxidative stress. Another finding was that Gapdh protein levels were decreased in dKO mice, a result that we also consistently observed in Obsl1-knockout cells and tissues (Supplementary Figs. 1b, 4a, 4b), necessitating the use of total protein, actin or myosin, for normalization. Most intriguingly, however, almost all identified mitochondrial electron transport chain proteins were downregulated in dKO mouse Sol and TA muscles (Fig. 6b, Supplementary Fig. 4c). Analysis of whole TA and/or Sol muscle extracts using the oxphos antibody cocktail or a Uqcrb antibody substantiated slightly or significantly decreased levels of mitochondrial complex I, II, III, IV, and V proteins in dKO mice (Fig. 6c, Supplementary Fig. 4d–f). Analysis of Prdx3 protein levels, a mitochondrially targeted peroxiredoxin family member^37^ that was also detected in our proteome analysis as significantly altered, indicating that the effect of Obsl1/obscurin loss on the mitochondria may go well beyond electron transport chain proteins (Fig. 6c, Supplementary Fig. 4e, f). Further analysis of the proteome data revealed that downregulation of electron transport chain proteins is more associated with loss of obscurin than Obsl1 (Supplementary Fig. 4c). However, only the loss of both proteins, obscurin and Obsl1, resulted in the substantial reduction in mitochondrial proteins. It remains to be demonstrated if these changes are mirrored on the physiological level, and if dKO muscles display altered reactive oxygen species (ROS) levels caused by mitochondrial insufficiency. Our proteome data showing changes to other peroxiredoxins, Sod2, and other proteins involved in ROS scavenging and signaling (Supplementary Fig. 5e; Supplementary Data File 1) are highly suggestive of this possibility.

## Discussion

Our study set out to investigate biological roles for Obsl1, with a specific focus on redundant functions between obscurin and Obsl1 for the development of skeletal muscles. For this, we generated conditional Obsl1-knockout mice using a strategy that prevents the expression of all Obsl1 isoforms. Surprisingly, our global Obsl1 knockouts display an embryonic lethality phenotype (Table 1).

Obsl1 has been shown to be responsible for ~20% of cases of 3M-growth syndrome, while mutations in cullin-7 and Ccdc8 account for the remainder of the patients^27,31^. Most of the Obsl1 patients suffer from mutations that result in a truncated protein due to frameshift mutations^31,38^. These findings led to the speculation that the Obsl1 mRNA in patients may be prematurely degraded by nonsense-mediated decay, resulting in complete absence of the protein^31^. However, similar to cullin-7 knockouts^39^, our global Obsl1 knockouts do not recapitulate the 3M-growth syndrome exhibited by patients.

Closer analysis showed that all Obsl1 mutations are located well 3′ of the sequence encoding for Ig domain 1 of Obsl1^31^. This leaves the potential open for low-level expression of C terminally truncated Obsl1 protein variants and alternative splice isoforms that circumvent some of the deleterious mutations in Obsl1, which is not possible in our mouse model. However, immunoblot analysis of Obsl1 using validated antibodies has not been extensively done on patient samples.

It is difficult to compare data from the 3M-growth syndrome transcriptome and the Obsl1 interactome^35,40^ with changes observed in our proteome analyses, as there are functional redundancies between Obsl1 and obscurin, which may not be present in skin fibroblasts and other non-muscle tissues (Supplementary Fig. 6a). Moreover, the complete dataset for the transcriptome analysis of fibroblasts from 3M-growth syndrome patients is not readily available^40^. However, there are several striking similarities between our proteome and the transcriptome and interactome datasets (Supplementary Fig. 5a, b; Supplementary Data File 5). Specifically, both studies found significant deregulation of genes/proteins involved in cellular transcription (e.g., Hnrnpf, Eef1a1, Ybx transcription factors), translation (small and large ribosomal subunits), and in genes involved in metabolic pathways downstream of IGF1 (insulin-like growth factor 1) and mTOR (mammalian target of rapamycin) signaling (Supplementary Fig. 5c). Several proteins that form part of membrane and vesicle trafficking complexes show up in both large datasets, including coatomer protein complex subunit alpha (Copa) or clathrin (Cltc) (Supplementary Fig. 5a, d). In addition, other proteins involved in vesicle and protein trafficking were found significantly altered, such as caveolin-3, muscle-related coiled-coil protein (MuRC/cavin-4)^41^, transferrin receptor (Tfrc)^42^, intersectin-1 (Itsn1)^43^, Rab8b^44^, non-clathrin-related endocytosis (pacsin3^45^, EHD3^46^), COP1- or COP2-related protein shuttling pathways (Sec31a^47,48^, archain-1 (Arcn1)^49^), and several tubulin isoforms, which were also deregulated in our proteome analysis (Supplementary Data 1). Deficiencies in several of these pathways have been implicated in growth retardation and developmental delays, including COP-related protein shuttling pathways^49–51^ or clathrin-dependent trafficking^52^. Another striking overlap between the datasets are proteins that are involved in metabolism, mitochondrial function (e.g., electron transport chain complex proteins), and generation/scavenging of ROS). Specifically, we found deregulation of Gapdh and Prdx3, both identified as putative interaction partners for Obsl1^35^ (Supplementary Figs. 5a, 6c). Maoa was also found downregulated in both the transcriptome and our proteome datasets. Patients with Maoa/Maob deficiency display loss of muscle tone and short statue^53,54^, while global Maoa/Maob dKO mice show developmental delays, behavior, and locomotion problems^55^. Closer examination of changes to muscles and exercise capacity in monoamine oxidase-deficient mice remains to be done.

It is thought that 3M-growth syndrome is caused by a deficiency in protein turnover due to the finding that a majority of patients with 3M-growth disorder display mutations in cullin-7, Obsl1, and Ccdc8. Indeed, Obsl1 was identified as a binding partner for the E3-ubiquitin ligase cullin-7^25,26,56^. However, our proteome data did not reveal significant changes to any of the other proteins associated with the disease, that is, cullin-7, Ccdc8, Fbw8, Igfbp2, or Igfbp5. These findings raise the possibility that functional deficiencies associated with the disorder may be tissue specific and spare skeletal muscles. Closer examination of expression patterns for 3M-growth syndrome associated proteins could reveal tissues types where this complex performs its major functions. Indeed, looking at expression profiles in Protein Atlas (https://www.proteinatlas.org/) reveals variable expression for Fbw8 (and many of the other F-box-containing substrate adaptor proteins for cullin-7), as well as Igfbp2 and Igfbp5 across tissues. The usage of cre lines that delete Obsl1 in tissues other than skeletal muscles and at earlier time-points will shed further light into non-muscle functions for Obsl1 and the development of 3M-growth syndrome.

Another surprise was the lack of baseline phenotype for skeletal muscle-specific Obsl1 knockouts, and the comparatively mild physiological phenotype observed for the dKO mice. Indeed, looking at the substantial molecular changes to the DSG complex, alterations to sarcolemmal integrity and to SR-associated proteins that the dKO mice display, it was unexpected to observe no changes to the number of centralized nuclei in muscle cross-sections as a sign for active muscle regeneration, and largely unchanged physiological twitch parameters. However, when investigating specific alterations to skeletal muscles upon loss of Obsl1 and obscurin redundant functions for both proteins start to emerge.

Despite extensive interactions of obscurin and Obsl1 (or its invertebrate homolog unc-89) with sarcomeric proteins^8,11,33,57^, loss of either or both obscurin family proteins had negligible effects on myofibrillogenesis and sarcomere structure. Indeed, apart from a slight reduction in the levels of myomesin-2 (Supplementary Fig. 1e), we were unable to detect any overt changes for myomesin-1, tropomyosin, α-actinin 2, titin-M8, and myosin localization or expression. These data indicate that obscurin and Obsl1 in vertebrate muscles are sarcomere-associated proteins that are not required for sarcomere assembly. Indeed, our experiments investigating muscle twitch characteristics only found increase in TtP values for loss of obscurin, indicative of problems with the SR and calcium-induced calcium release. However, no changes were found in other parameters measured in these experiments, with the exception of stress during tetanus values for obscurin knockouts. Obsl1 has been shown to target to several subcellular localizations in heart and skeletal muscles, including M-band, Z-disc, and intercalated discs^8,11^. While not all antibodies in these studies have been verified against the knockout, our experiments using two verified Obsl1 antibodies that target different Obsl1 epitopes corroborate several of these findings. Known and novel interactions may explain the differential Obsl1 localization to sarcomeric M-bands and Z-discs (Fig. 1c, Supplementary Figs. 1a, 1d, 1e, 6b). It remains to be demonstrated how splice variants of Obsl1, and binding affinities to interactors modifies the subcellular localization.

Obscurin knockouts display altered ankyrin-B and dystrophin localization at costameres, and increased membrane fragility combined with reduced muscle exercise tolerance^13,14^. Morpholino-mediated knockdown of obscurin-A in zebrafish also resulted in abnormal dystrophin and α-dystroglycan localization, suggesting evolutionary conserved functions of obscurin for DSG complex organization and sarcolemmal integrity^58^. Following these studies, we investigated expression and localization of DSG components and found changes at baseline predominantly in dKO mice. While dKO muscles did not display changes to dystrophin levels, its subsarcolemmal localization appeared patchy and its distribution disturbed. Immunofluorescence imaging of subsarcolemmal dystrophin indicated that obscurin and Obsl1 may partially modulate the membrane targeting of dystrophin, and help it to spread evenly over the sarcolemmal membrane (Fig. 3b, Supplementary Fig. 2b).

Muscles of dKO mice also displayed a surprising slight decrease in utrophin (*p* = 0.06), and several other investigated DSG components, including α-dystrobrevin. Moreover, DSG-linked signaling via ion channels (Trpc1) may also be altered in dKO mice only, based on reduced expression levels of this channel. These data suggest that both Obsl1 and obscurin have redundant functions for DSG complex assembly and stability. Loss of both proteins resulted in impaired sarcolemmal integrity in a substantial proportion of muscles in mice at baseline (not exercised), as demonstrated by positive immunoglobulin labeling of muscle fibers in dKO mice (Fig. 3c, Supplementary Fig. 2c).

Muscles of dKO mice also show upregulation of proteins involved in muscle repair processes, including Dysf and FlnC^59–61^. Future experiments that further characterize membrane damage and studies that investigate repair mechanisms through FlnC and Dysf should clarify the extent of the sarcolemmal integrity impairment in dKO mice.

While some of the functions that obscurin exerts on the DSG complex are due to interactions with ankyrin isoforms, spectrin and FlnC^13,20,21,62,63^, we and others identified that Obsl1 may interact with other proteins linked to the DSG complex and its associated components^35^, including FlnC, tubulin, dystonin, plectin, ahnak, and utrophin (Supplementary Figs. 1a, 5a, 6c). Obsl1/obscurin function has also been implicated for microtubule integrity, by promoting microtubule assembly and/or stability^13,14,64^. Microtubule action is important for SR organization^65^, sarcolemmal integrity and repair^66,67^, cell division, cellular shape, and the transport of vesicles over longer distances, in addition requiring actions of kinesin and dynein as molecular motors^68^. When looking at the proteome data, we noticed increases in α- and β-tubulins, which can be verified in immunoblot analyses of dKO TA muscles (Fig. 3a, Supplementary Fig. 2a). Alterations in the microtubule network have also been linked to the development of muscular dystrophy in *mdx* mice^69^. Dystrophin was shown to interact with microtubules^70,71^, and its loss increased the amounts of both α- and β-tubulins^69^, a finding that is similar to what is seen in our dKO mice.

One of the key findings in skeletal muscles of obscurin knockouts was the importance of the protein for SR architecture through its link with the small muscle-specific ankyrin-1 isoform sAnk1.5 that is embedded in the SR membrane^20,21^. While loss of Obsl1 has little or no impact on protein levels of SR-associated proteins, muscles of dKO mice displayed profound alterations to levels of lumenal SR calcium-binding proteins sarcalumenin and Casq2, as well as Serca. Our proteome and expression data also indicated increases to junctional SR proteins like triadin, junctin, or RyRs in dKO muscles, something not previously observed for obscurin knockouts^12^. One might speculate based on the predominant subcellular localizations of obscurin at the M-band and Obsl1 at the M-band and Z-disc that both the longitudinal and junctional SR are affected in dKO muscles. Data on DSG-associated ion channels (Trpc1), the junctional (RyR) or longitudinal SR membranes (Serca), suggests that loss of obscurin/Obsl1 alters calcium handling in dKO muscles. These data in combination with published findings on Speg^17^ and the invertebrate homolog unc-89^72^ substantiates the finding that the regulation of SR architecture and function is a major biological role for all obscurin protein family members.

Many questions remain unanswered as to the exact molecular origins for the observed effects in dKO muscles. Our proteome analyses may provide clues for some of the molecular mechanisms at play that could result in alterations to the cellular metabolism, changes to membrane stability and repair mechanisms, and the impact on calcium handling and Serca function/regulation. However, none of the well-characterized muscle-specific obscurin and Obsl1 interaction partners may account for all of the observed phenotypical changes^2,11,20,21,25,26,57,73^. Indeed, it remains to be demonstrated for the majority of the more than 600 identified binding partners of obscurin and Obsl1^35^, whether they play a role in muscles, and how loss of both obscurin protein family members affect their expression, localization, and cellular functions.

## Methods

### Gene targeting and generation of Obsl1-knockout mice, analysis of animal physiology

The targeting construct was generated by subcloning isogenic 129svj genomic DNA fragments of the murine *Obsl1* gene into a targeting vector, and placing coding exons 1 to 4 between loxP sites (Supplementary Fig. 1a). The construct was linearized, and electroporated into R1 embryonic stem cells. G418-resistant clones were screened for correct homologous recombination by Southern blot analysis described elsewhere^12^. Germline transmission was tested by appearance of the agouti fur color phenotype of 129-derived ES-cells, after which animals were backcrossed into the black swiss background. Genotying was done by PCR using oligonucleotides shown in Supplementary Table 1. Removal of neomycin cassette was done by crossing floxed Obsl1 mice with mice carrying Flpase recombinase^74,75^. Generation of global Obsl1 knockouts was done by crossing floxed Obsl1 mice with mice carrying Cre recombinase under control of the protamine promoter^30^. Finally, generation of skeletal muscle-specific Obsl1 knockouts was achieved by crossing floxed Obsl1 mice with mice carrying Cre recombinase under control of the myogenin promoter^32^. Successful recombination of *Obsl1* gene and generation of skeletal muscle knockouts was validated by immunoblot analyses (Fig. 1a). Generation of obscurin/Obsl1 myogenin-cre dKOs was done by crossbreeding into the obscurin knockout background^12^. If not explicitly stated, all investigations were done with tissues from animals of both sexes. Experiments using the fifth toe of the EDL muscle from 4-month-old mice was done as described previously^12^. All procedures involving genetically modified animals have been approved by the UC San Diego institutional oversight committee.

### Cell culture

Culture of cos-1 cells was done as previously described^76,77^. To test Obsl1 antibodies and to determine if effects seen in dKO and Obsl1-knockout muscles are also seen in non-muscle Obsl1-knockout cells, we isolated cells from lungs of heterozygous global Obsl1 knockouts (+/−) and from mice that have a knockout and a floxed allele for Obsl1 (flox/−), using a collagenase/dispase enzyme mix (Roche). Isolated cells were cultured and propagated in growth medium (10% fetal calf serum, 1% penicillin/streptomycin, Dulbecco’s modified Eagle’s medium [Corning]). To generate Obsl1 knockouts, cells were transduced with a lentivirus carrying a cre-blasticidin-RFP expression cassette under control of a CMV promoter (#LVP013; Gentarget Inc.), allowing for recombination of the Obsl1-floxed allele and selection of transduced cells by 5 µg/ml blasticidin (Thermo Fisher). After transduction, cells were kept in growth medium supplemented with blasticidin until harvest for analysis of proteins.

### Protein analysis, immunofluorescence, histology, and antibodies

For analysis of protein levels, muscles or cells were lysed directly into sample buffer (3.7 M urea, 134.6 mM Tris-HCl, pH 6.8, 5.4% sodium dodecyl sulfate (SDS), 2.3% NP-40, 4.45% β-mercaptoethanol, 4% glycerol, and 6 mg/100 ml bromophenol blue). Loading was normalized by densitometry of Coomassie-stained gels using either actin or myosin bands. Once normalized, muscle lysates were loaded onto SDS-polyacrylamide gel electrophoresis (PAGE) gels (8, 12, or 15% acrylamide concentration) or SDS-Agarose gels^12^. Proteins were transferred onto nitrocellulose membranes, and detection of proteins was done using antibodies as described elsewhere^77^. If not stated otherwise, biological replicates were used for immunoblot analyses. Sample sizes for quantification of protein levels are given in the figure or figure legend. Uncropped blot images for all western blot data are presented in Supplementary Data File 6.

For immunofluorescence, muscles were isolated, snap frozen while submerged in isobutane, and embedded into cryogenic molds using optimal cutting temperate (OCT) medium. Longitudinal or cross-sections of muscles were done by cryosectioning using a cryostat (Leica). Sections were collected on surface-treated microscopy glass slides (Colorfrost Plus, Fisher), dried, and stored at −80 °C until further use. Immunofluorescent staining of sections was done by fixing sections in ice-cold acetone (5 min at −20 °C), rehydration of tissue using 1× phosphate-buffered saline (PBS) (5 min), permeabilization (1× PBS, 0.2% Triton X-100; 5 min), and blocking with 1% bovine serum albumin (BSA) fraction V and 5% normal donkey serum diluted in Gold Buffer (155 mM NaCl, 2 mM EGTA, 2 mM MgCl_2_, 20 mM Tris-HCl, pH 7.5) for 30 min. After blocking of non-specific binding sites, tissue sections were incubated with primary antibody diluted in Gold Buffer overnight at 4 °C. Following three washes with PBS (5 min each), sections were incubated with fluorescently labeled secondary antibodies combined with either 4′,6-diamidino-2-phenylindole (DAPI) and/or fluorescently labeled wheat germ agglutinin (WGA) (Sigma) diluted into Gold Buffer for 2 h at room temperature. After washing with 1× PBS (5 min each), sections were embedded using fluorescent mounting medium (DAKO), and covered with coverslips. Immunofluorescently labeled tissues were imaged using a Fluoview 1000 confocal microscope (Olympus), in sequential scanning mode using ×10 air or ×40 oil objectives and zoom rates between 1× and 3×. For analysis of cross-sectional areas, images were analyzed using the area-measuring tool in ImageJ.

Primary antibodies used in this study are listed in Supplementary Table 2. If not noted otherwise, all secondary antibodies were either from DAKO or Jackson ImmunoResearch. Fluorescently labeled WGA was obtained from Invitrogen/Thermo Fisher. DAPI was purchased from Sigma-Aldrich.

### Mass spectrometry and proteome analysis

Proteins were isolated form whole muscle and lysed into ice-cold isolation buffer (300 mM KCl, 30 mM PIPES pH 6.6, 0.5% NP-40, 1× protease inhibitor (Roche), 1× Phos-stop (Roche)). Insoluble proteins were removed by centrifugation (14,000 rpm, 10 min at 4 °C), and the supernatant was diluted 1:4 with ice-cold dilution buffer (1× Phos-stop (Roche), 0.5% NP-40, 1 mM dithiothreitol (DTT)). Precipitation of acto-myosin components was done by centrifugation (14,000 rpm, 15 min at 4 °C), and the remaining supernatant snap frozen for further analysis by mass spectrometry and immunoblot analyses.

Analysis and identification of peptides via mass spectrometry was done as described previously^78^. Briefly, immediately prior to mass spectrometry, protein solutions were diluted in TNE buffer (50 mM Tris-HCl, pH 8.0, 100 mM NaCl, 1 mM EDTA). RapiGest SF reagent (Waters Corp.) was added to the mix to a final concentration of 0.1% and samples were boiled for 5 min. TCEP (Tris (2-carboxyethyl) phosphine) was added to a final concentration of 1 mM, and the samples were incubated at 37 °C for 30 min. Subsequently, the samples were carboxymethylated with 0.5 mg/ml of iodoacetamide for 30 min at 37 °C, followed by neutralization with 2 mM TCEP (final concentration). Protein samples prepared as above were digested with trypsin (trypsin:protein ratio of 1:50) overnight at 37 °C. RapiGest was degraded and removed by treating the samples with 250 mM HCl at 37 °C for 1 h, followed by centrifugation at 14,000 rpm for 30 min at 4 °C. The soluble fraction was then added to a new tube and the peptides were extracted and desalted using C18 desalting tips (Thermo Scientific).

The trypsinized samples (eight samples) were labeled with isobaric tags (iTRAQ8, ABSCIEX)^79^, where each sample was labeled with a specific tag to its peptides as described in the manufacturer’s instructions. Each set of experiments were then pooled and fractionated using high pH reverse phase chromatography (HPRP-Xterra C18 reverse phase, 4.6 mm × 10 mm, 5 µm particle [Waters]). The chromatography conditions were as follows: the column was heated to 37 °C and a linear gradient from 5 to 35% B (Buffer A—20 mM ammonium formate pH 10 aqueous, Buffer B—20 mM ammonium formate pH 10 in 80% acetonitrile (ACN)-water), followed by 5 min at 100% B was applied for 80 min at 0.5 ml/min flow rate. A total of 42 fractions of 0.5 ml volume were collected. For liquid chromatography with tandem mass spectrometry (LC-MS/MS) analysis, some fractions were pooled to create a final 16 pooled samples. Each of the pooled fractions were analyzed by high pressure liquid chromatography coupled with LC-MS/MS using nano-spray ionization.

Nano-spray ionization experiments were performed with a TripleTof 5600 hybrid mass spectrometer (ABSCIEX) interfaced with nano-scale reversed-phase UPLC (Waters Corporation, nanoACQUITY) using a 20 cm × 75 µm ID glass capillary packed with 2.5 µm C18 (130) CSHTM beads (Waters Corporation). A linear gradient (5–80%) of ACN was used to elute the peptides from the C18 column into the mass spectrometer at a flow rate of 250 μl/min for 1 h. The ACN gradient was created with the following buffers: Buffer A (98% H_2_O, 2% ACN, 0.1% formic acid, and 0.005% trifluoroacetic acid (TFA)) and Buffer B (100% ACN, 0.1% formic acid, and 0.005% TFA). MS/MS data were acquired in a data-dependent manner in which the MS1 data was acquired for 250 ms at *m*/*z* of 400 to 1250 Da and the MS/MS data was acquired from *m*/*z* of 50 to 2000 Da. Independent data acquisition parameters were as follows: MS1-TOF (mass spectrometry imaging-time of flight) acquisition time of 250 ms, followed by 50 MS2 events of 48 ms acquisition time for each event. The threshold to trigger MS2 event was set to 150 counts when the ion had the charge state +2, +3, and +4. The ion exclusion time was set to 4 s. The collision energy was set to iTRAQ experiment setting. Finally, the collected data were analyzed using Protein Pilot 5.0 (ABSCIEX) for peptide identifications and Peaks^80^. Bioinformatic enrichment and pathway analysis was done using Metascape (http://metascape.org/)^81^, Morpheus (https://software.broadinstitute.org/morpheus/), the BioGRID (https://thebiogrid.org/)^82^, and Venny (http://bioinfogp.cnb.csic.es/tools/venny).

### Eukaryotic expression constructs, yeast two-hybrid screening, and co-immunoprecipitation

Constructs for green fluorescent protein (GFP)-tagged Obsl1 fragments and yeast two-hybrid screening were generated as described elsewhere^24^. In short, coding sequences for human Obsl1 fragments (NCBI access no: NM_178884) were subcloned to enhanced GFP (EGFP) (Clontech) or pLex vectors to produce in-frame fusion between GFP or the DNA-binding domain and the insert. All expression constructs were sequenced to determine correct integration into the vector. The yeast two-hybrid screening was done as described previously^77^. Briefly, L40 yeasts were transformed with bait constructs (pLex-Obsl1 domain truncations) and empty prey vector (pAct2) to perform auto-activation test. pLex-Obsl1 constructs that were negative for auto-activation were selected for further yeast two-hybrid screening. L40 yeasts were transformed with selected pLex-Obsl1 constructs, and positive transformants were used to perform a library transformation. Yeasts were plated on selective dropout plates, and colonies were allowed to form for 5 days. Yeast clones positive for β-galactosidase activity in an X-gal overlay assay were further amplified, and library plasmid DNA was extracted and analyzed by sequencing.

Transfection of Cos-1 cells was done as described previously^77^. Transfected cells were lysed into ice-cold immunoprecipitation (IP) buffer (100 mM NaCl, 10 mM Tris-HCl, pH 8, 1× Complete Protease Inhibitor Cocktail [Roche], 1 mM DTT, and 0.5% NP-40) 2 days after transfection. Lysates were briefly sonicated, centrifugated for 10 min at 14,000 rpm (at 4 °C) to remove insoluble proteins, and supernatants were used to perform immunoprecipitation. Briefly, 5 µg GFP antibody (Roche) were added to lysates, and incubated overnight at 4 °C to allow for formation of immunocomplexes. Subsequently, magnetic protein-G-coated beads (Dynabeads, Life Technologies) were added and incubated for 3 h at 4 °C on a shaker. Bound protein immunocomplexes were separated from unbound proteins by magnetic separation, and the beads were washed three times with ice-cold wash buffer (1× PBS, 0.2% NP-40). Analysis of input and bound protein immunocomplexes was done by SDS-PAGE, followed by immunoblot analysis.

### Bioinformatics and statistical analysis

The following bioinformatics packages and programs were used to perform analyses in this manuscript: analysis of proteome data was done using Peaks Studio (http://www.bioinfor.com/peaks-studio/)^80^; pathway enrichment analysis was done with the help of MetaScape (http://metascape.org/)^81^; hierarchical clustering of proteome data was done using Morpheus (https://software.broadinstitute.org/morpheus/); image analysis was done using ImageJ with the LOCI BioFormats and Image5D plugins (by the Open Microscopy Environment consortium and Joachim Walter, respectively); sequence alignments and domain structure analysis were done using NIH/NCBI BLAST (https://blast.ncbi.nlm.nih.gov/Blast.cgi), SDSC Biology Workbench (http://workbench.sdsc.edu/), and SMART (http://smart.embl-heidelberg.de/)^83^. With the exception for built-in statistics by the Peaks Studio software, statistical analysis of all data was done by using analysis of variance comparison, followed by Dunnett’s multiple comparisons test, or unpaired *t* test, performed using Excel (Microsoft) or GraphPad Prism version 7 for Mac (GraphPad Software, http://www.graphpad.com). Violin plots were generated using BoxPlotR (http://shiny.chemgrid.org/boxplotr/). Results are presented as means ± standard error. *P* values of *p* < 0.05 were considered statistically significant. Sample sizes are indicated in the figure or figure legend. If not stated otherwise, both sexes were analyzed in the experimental procedures. We used biological replicates to showcase biological variability and reproducibility of findings. Whenever appropriate, different methods were used to verify a finding (e.g., interaction found in yeast two-hybrid screening was verified by co-immunoprecipitation). No data were excluded in the generation of figures/tables, with the exception of two lanes in Supplementary Fig. 7b (upper panel) that did not pass the quality control as judged by Ponceau stain.

### Reporting summary

Further information on experimental design is available in the Nature Research Reporting Summary linked to this article.

## Supplementary information

Supplementary Information

Description of Additional Supplementary Files

Reporting Summary

Supplementary Data 1

Supplementary Data 2

Supplementary Data 3

Supplementary Data 4

Supplementary Data 5

Supplementary Data 6

## Data Availability

All relevant data described in the manuscript can be found in the figures, tables, and supplemental files, are deposited in public repositories, or can be requested from the authors. The mass spectrometry proteomics data have been deposited to the ProteomeXchange Consortium via the PRIDE partner repository^84,85^ with the dataset identifier PXD013008 and 10.6019/PXD013008.
