# Supplementary material for: Murine obscurin and Obsl1 have functionally redundant roles in sarcolemmal integrity, sarcoplasmic reticulum organization, and muscle metabolism
Source: Commun Biol. 2019 May 9;2:178. doi: 10.1038/s42003-019-0405-7 (PMC6509138; doi:10.1038/s42003-019-0405-7)
Supplement: Supplementary file 1 — Supplementary Information [file 42003_2019_405_MOESM1_ESM.pdf]

Supplementary Figures and Tables

Supplementary Figure 1

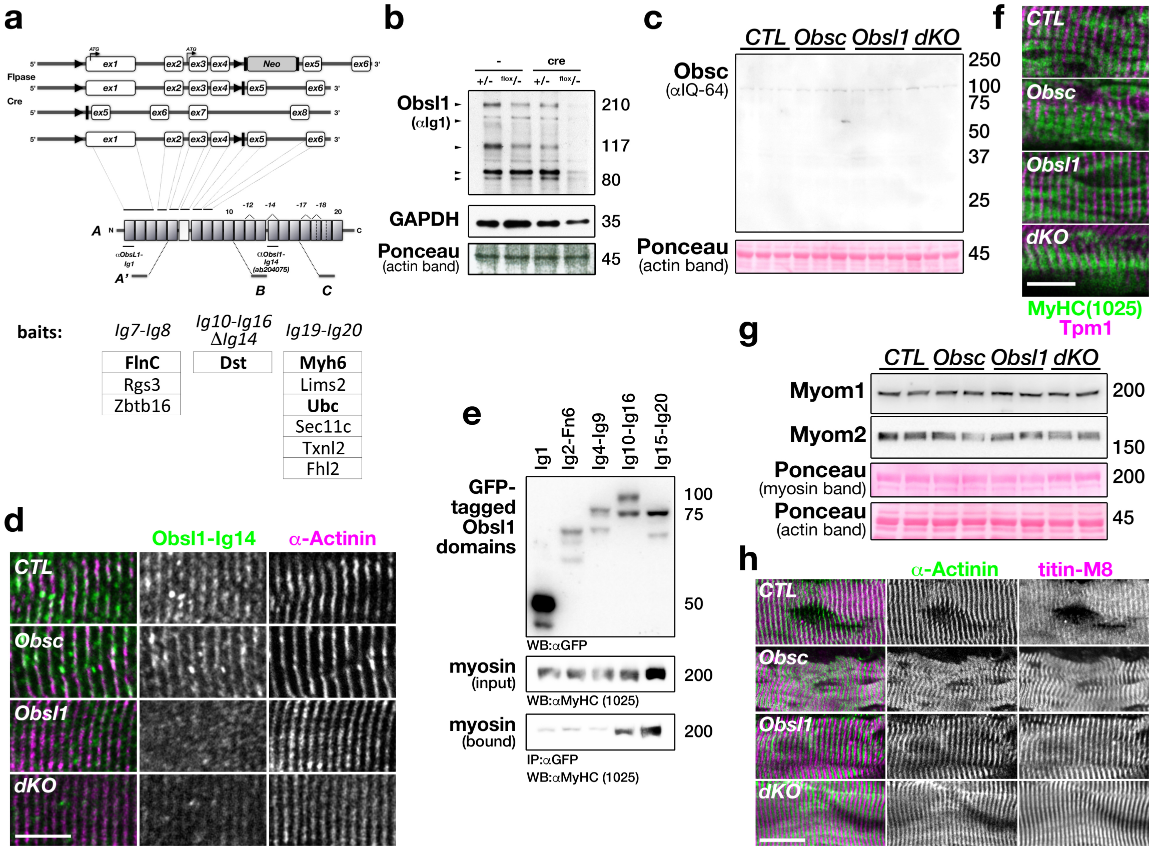

Supplementary Figure 1. (a) Obsl1 knockout strategy, splice variants, domain layout, epitopes of antibodies used in this study, and novel interactions identified by yeast-two hybrid assay. Exons (ex), start codons, locations of LoxP (black arrowheads) and FRT sites (black rectangle), as well as the neomycin cassette (Neo) are indicated. Used bait constructs (Obsl1-Ig7-Ig8, Obsl1-Ig10-Ig16ΔIg14, Obsl1-Ig19-Ig20) and identified putative novel interaction partners are shown. Binding partners verified by this study or also identified by an independent method35 are highlighted in bold. (b) Verification of Obsl1 knockout strategy using lung endothelial cells isolated from heterozygous Obsl1 animals (+/-) or heterozygous floxed Obsl1 animals (flox/-). Immunoblot analysis of Obsl1 expression using the Obsl1-Ig1 antibody and whole cell lysates from control cells (-) and cells transduced with a lentivirus carrying a cre-blasticidin-RFP expression cassette (cre) under control of a CMV promoter. GAPDH expression levels for each cell-line are also shown. Ponceau stained actin band is shown as loading control. (c) Analysis of whole TA muscle extracts for expression of small obscurin isoforms (<250 kDa). Lysates from control (CTL), obscurin knockout (Obsc), skeletal muscle specific Obsl1 (Obsl1) and double knockout (dKO) muscles show a weak band at approx. 100kDa, which does not change between the groups. Ponceau stained actin band is shown as loading control. (d)

Sarcomeric localization of Obsl1 (Ig14 epitope antibody) in Obsc, Obsl1, dKO as well as control (CTL) TA muscles. Please note that some background staining caused by non-specific cross-reactivity of the antibodies is observed in knockout tissues. Scale bar = 10µm.(e) Co-immunoprecipitation of GFP-tagged Obsl1 domain truncations with sarcomeric myosin (clone 1025). (f) Immunofluorescence analysis of sarcomeric myosin (MyHC, clone 1025; green) localization in frozen sections of TA muscles from control (CTL), obscurin knockout (Obsc), skeletal muscle specific Obsl1 knockout (Obsl1) and double knockout (dKO) mice. Tropomysin (TPM1) was used as a counterstain (magenta). Scale bar = 10µm. (g) Immunoblot analysis of myomesin (Myom1) and M-protein (Myom2) expression levels in CTL, Obsc, Obsl1 and dKO TA muscles. Ponceau stained actin and myosin bands are shown as loading controls. (h) Immunofluorescence analysis of sarcomeric alpha-actinin 2 and titin-M8 localization in frozen sections of TA muscles from control (CTL), obscurin knockout (Obsc), skeletal muscle specific Obsl1 knockout (Obsl1) and double knockout (dKO) mice. Scale bar = 20µm.

Supplementary Figure 2

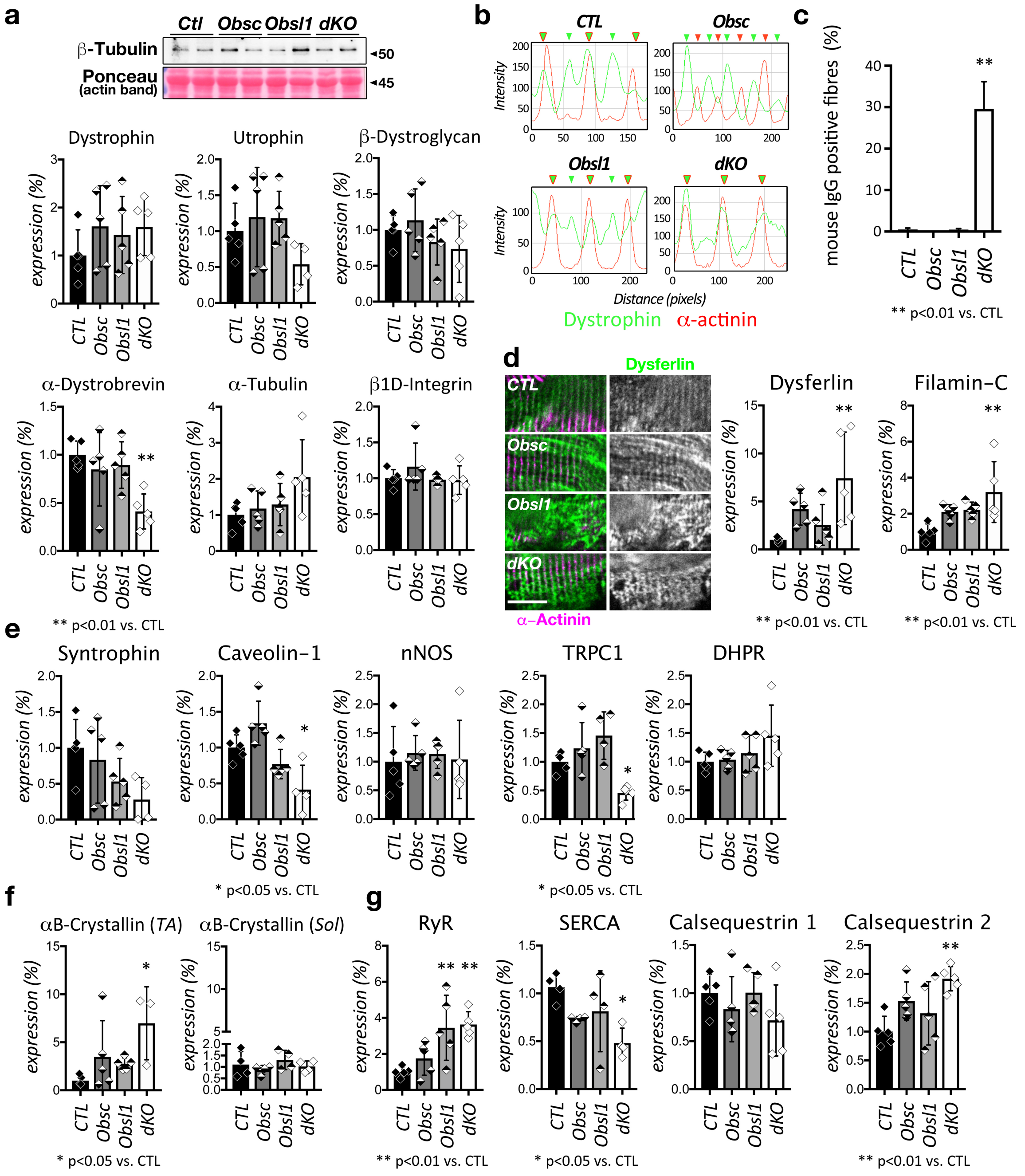

Supplementary Figure 2. (a) Expression levels of beta-tubulin in whole protein lysates of TA muscles of control (CTL), obscurin knockout (Obsc), Obsl1 skeletal muscle knockout (Obsl1) and double knockout (dKO) mice (top panel). Ponceau stained actin band is shown as loading control. Quantification of dystrophin-sarcoglycan complex protein expression levels (bottom panel). ** $p<0.01$ vs. CTL as determined by ANOVA. (b) Analysis of subsarcolemmal dystrophin localization (green) as determined by RGB-plot

(ImageJ) in TA muscles of CTL, Obsc, Obsl1 and dKO mice (from Figure 3b). Localization of the Z-disc was determined by sarcomeric alpha-actinin 2 counterstain (red). Note that profile in dKO muscle was determined in areas where dystrophin was observable. (c) Quantification of mouse IgG positive muscle fibres from TA muscle of all groups (see also Figure 3c). More than 125 fibres were scored for each group (n). ** $p<0.01$ vs. CTL. (d) Immunofluorescence analysis of dysferlin localization in frozen sections from TA muscles of CTL, Obsc, Obsl1 and dKO mice (left panel). Scale bar=10μm. Quantification of dysferlin and filamin-C expression levels in TA muscles of CTL, Obsc, Obsl1 and dKO mice (right panels). ** $p<0.01$ vs. CTL as determined by ANOVA. (e) Quantification of syntrophin, caveolin-1, nNOS, Trpc1 and DHPR alpha-2 subunit expression levels in TA muscles of CTL, Obsc, Obsl1 and dKO mice. * $p<0.05$ vs. CTL as determined by ANOVA. (f) Quantification of alphaB-crystallin expression levels in TA (left panel) or Sol muscles (right panel) of CTL, Obsc, Obsl1 and dKO mice. * $p<0.05$ vs. CTL as determined by ANOVA. (g) Quantification of RyR, Serca, Calsequestrin 1 and Calsequestrin 2 expression levels in TA muscles of CTL, Obsc, Obsl1 and dKO mice. * $p<0.05$ vs. CTL, ** $p<0.01$ vs. CTL as determined by ANOVA. (a, d-g) Sample size (n) is indicated in the figure. Immunoblots used to quantify protein expression levels are shown either in Figures 3-5 or Supplementary Figure 7.

Supplementary Figure 3

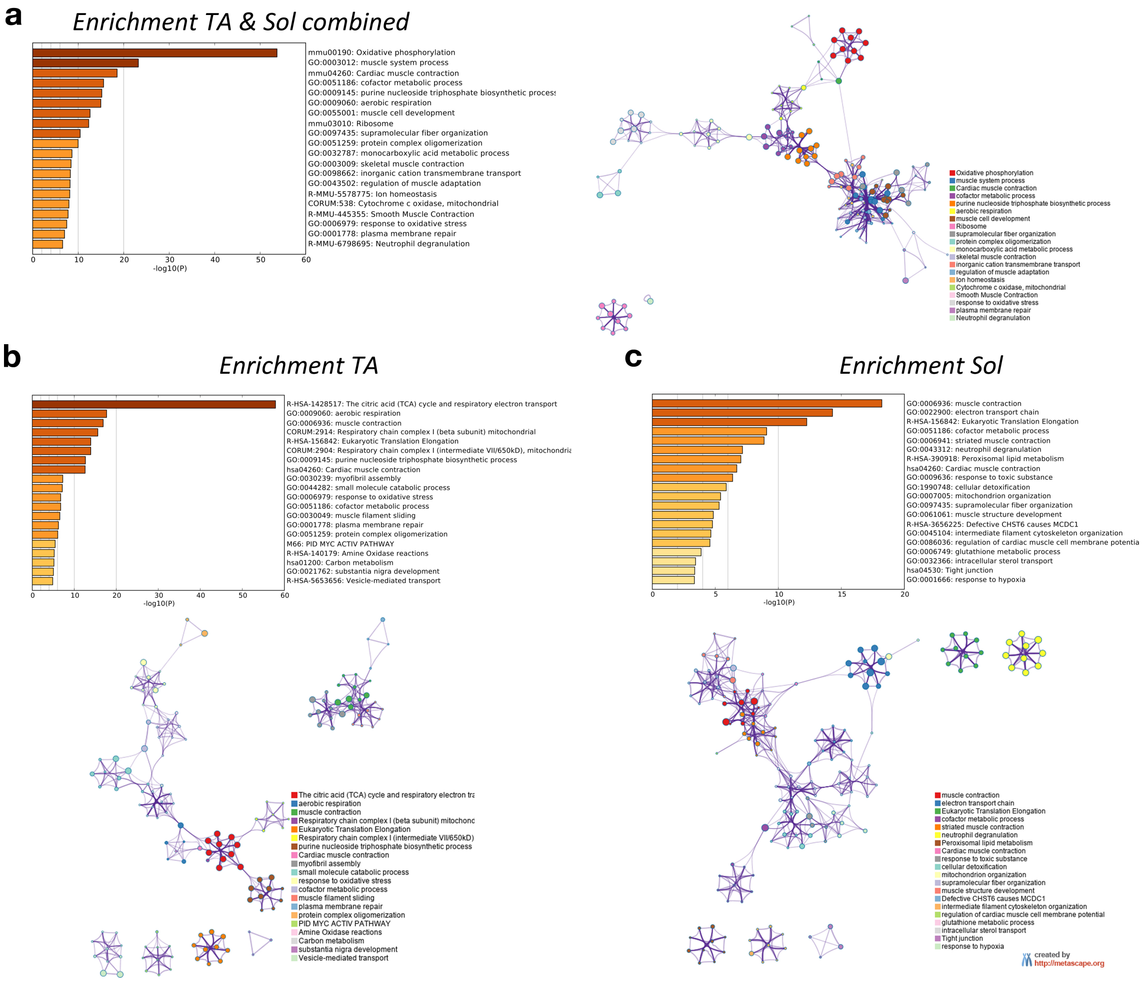

Supplementary Figure 3. Enrichment and pathway analysis of significantly changed proteins in tibialis anterior (TA) and soleus (Sol) muscles in combination (a) or separately (b: TA only; c: Sol only) using Metascape 81. Additional information on the pathway enrichment analysis can be found in Supplementary Data Files 1-4.

Supplementary Figure 4

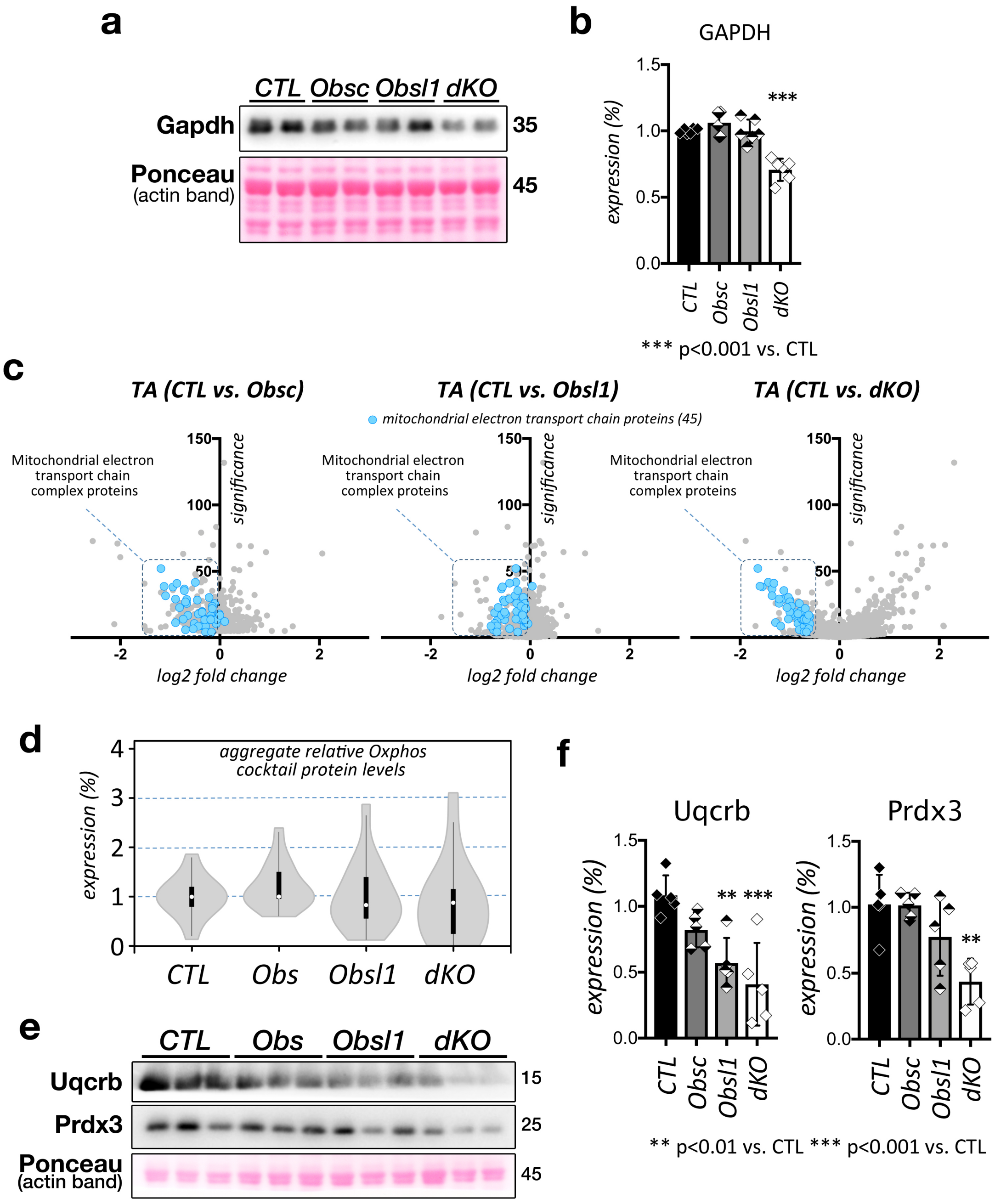

Supplementary Figure 4. Analysis of changes to metabolic enzymes and mitochondrial electron transport chain complex proteins. (a, b) Immunoblot analysis (a) and quantification (b) of GAPDH protein levels in tibialis anterior (TA) muscles of control (CTL), obscurin knockout (Obsc), skeletal muscle specific Obsl1 knockout (Obsl1) and double knockout (dKO) mice. Ponceau stained actin band is shown as loading control. Sample size (n) and p-values as determined by ANOVA are indicated in the figure. (c)

Volcano plot analysis of changes to electron transport chain complex proteins in TA muscles of Obsc vs. CTL (left panel), Obsl1 vs. CTL (middle panel) and dKO vs. CTL (right panel). (d) Violin plots of aggregate oxphos antibody quantifications in TA muscles. Sample size for oxphos antibody analysis was n=4. White circles show the medians; box limits indicate the 25th and 75th percentiles as determined by R software; whiskers extend 1.5 times the interquartile range from the 25th and 75th percentiles; polygons represent density estimates of data and extend to extreme values. (e, f) Immunoblot analysis (e) and quantification (f) of peroxiredoxin-3 (Prdx3) and Uqcrb protein levels in tibialis anterior (TA) muscles of control (CTL), obscurin knockout (Obsc), skeletal muscle specific Obsl1 knockout (Obsl1) and double knockout (dKO) mice. Ponceau stained actin band is shown as loading control. Sample sizes (n) and p-values as determined by ANOVA are indicated in the figure.

Supplementary Figure 5

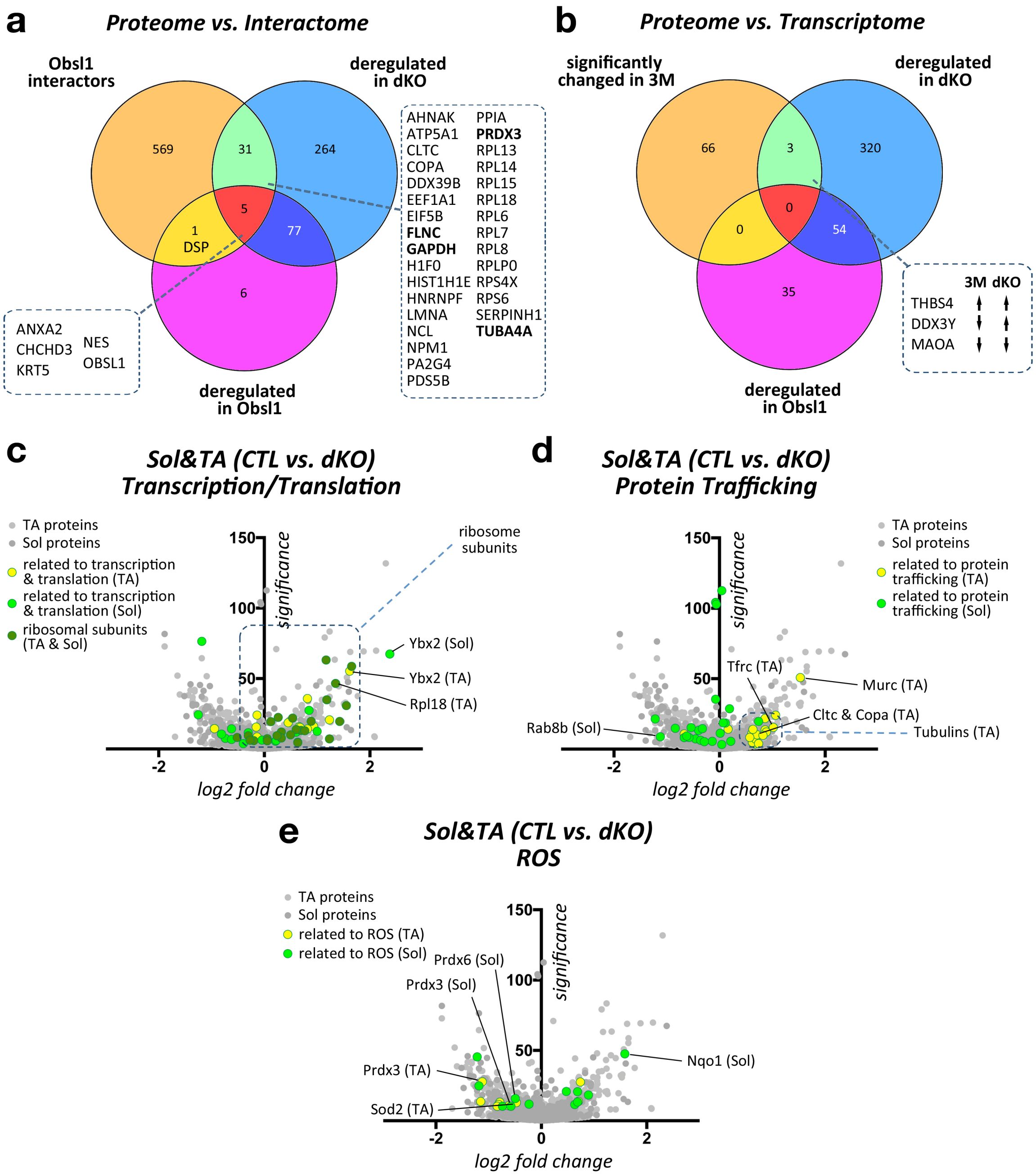

Supplementary Figure 5. (a, b) Comparison of significantly changed proteins identified in the proteome data from skeletal muscle specific Obsl1 knockout (Obsl1) or obscurin-Obsl1 double knockout mice (dKO) with known and novel Obsl1 interaction partners35 (a) or significantly changed transcript identified in 3M-growth syndrome patients40 (b). Proteins that overlap in both datasets are shown. Proteins highlighted in bold were investigated/verified in this study. (c-e) Volcano plot of significantly changed proteins between control (CTL) and dKO tibialis anterior (TA) and soleus (Sol) muscles involved in

gene-regulation by transcription or translation (c), protein trafficking (d) and reactive oxygen species (ROS) signaling and scavenging (e).

Supplementary Figure 6

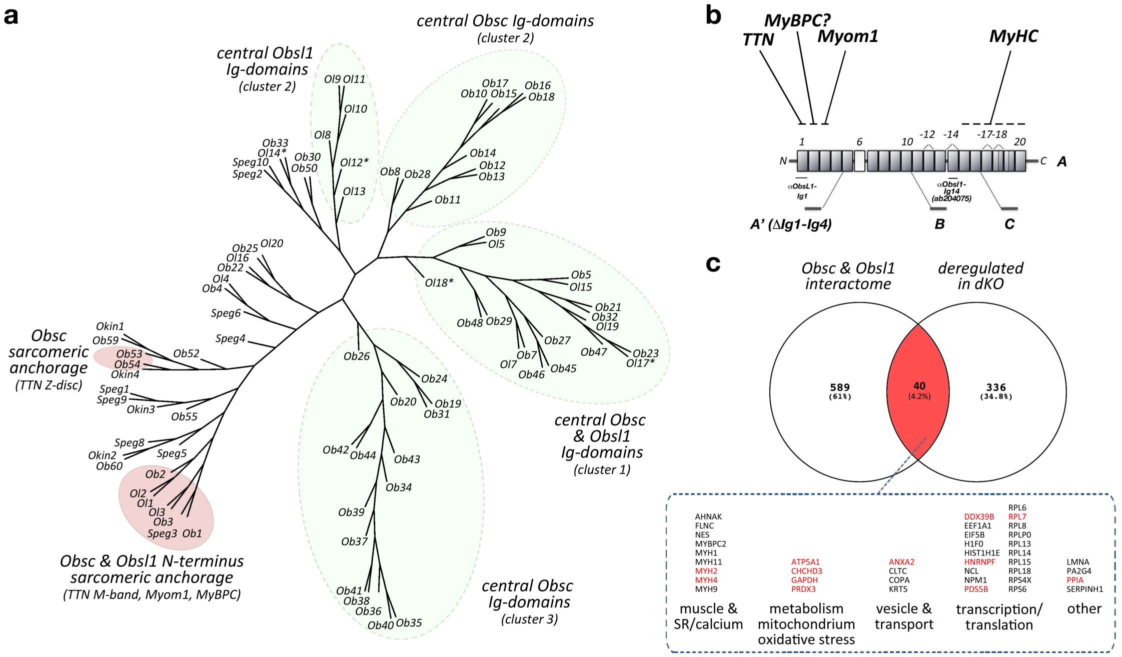

Supplementary Figure 6. (a) Sequence alignment dendrogram of Ig-domains from human obscurin, Obsl1 and SPEG. Clusters of highly related Ig-domain sequences are highlighted, as are binding partners for obscurin and Obsl1. Asterisks identify Ig domains in Obsl1 that may be spliced out in some isoforms of the protein. This includes Ig-domain 14 that is recognized by one of the antibodies used in this study. (b) Schematics of Obsl1 domain layout, splice isoforms, location of antibody epitopes and minimal mapped binding sites for suspected, known and novel myofilament interaction partners. (c) Comparison of the obscurin and Obsl1 interactome (see also Supplementary Data File 5) with proteins found deregulated in tibialis anterior and soleus dKO muscles. Proteins that are found in both groups are shown in the box below. Downregulated proteins are marked in red.

Supplementary Figure 7

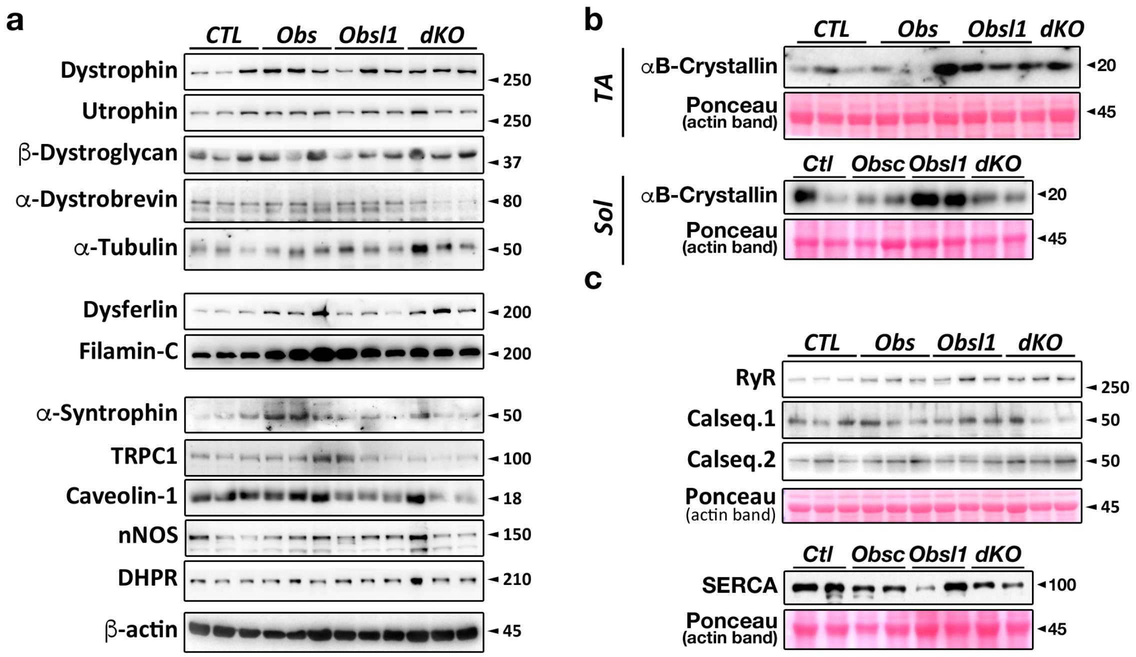

Supplementary Figure 7. (a-c) Immunoblot analysis of dystrophin-sarcoglycan complex associated proteins (a), alphaB-crystallin (b) and SR-associated proteins (c) in total muscle lysates from control (CTL), obscurin knockout (Obsc), skeletal muscle specific Obsl1 knockout (Obsl1) and double knockout (dKO) mice. All lysates were from TA muscles, except when indicated in the figure.

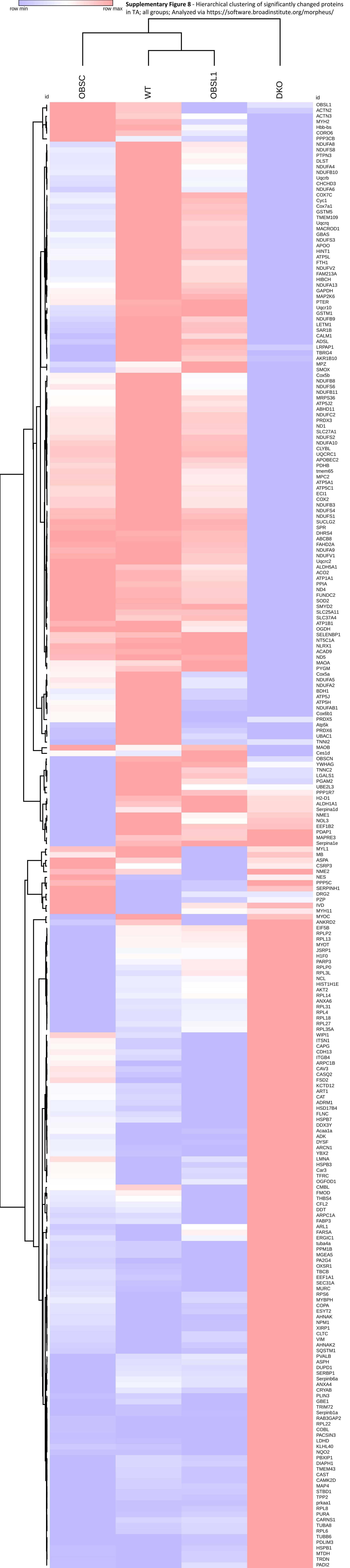

Supplementary Figure 8 - Hierarchical clustering of significantly changed proteins in TA; all groups; Analyzed via https://software.broadinstitute.org/morpheus/

Supplementary Figure 8. Hierarchical clustering of significantly altered proteins identified in tibialis anterior (TA) muscles of control (CTL), obscurin knockout (Obsc), skeletal muscle specific Obsl1 knockout (Obsl1) and double knockout (dKO) mice using Morpheus. Data from this analysis has been used to generate Figure 4a.

Supplementary Figure 9 - Hierarchical clustering of significantly changed proteins in Sol; all groups; Analyzed via https://software.broadinstitute.org/morpheus/

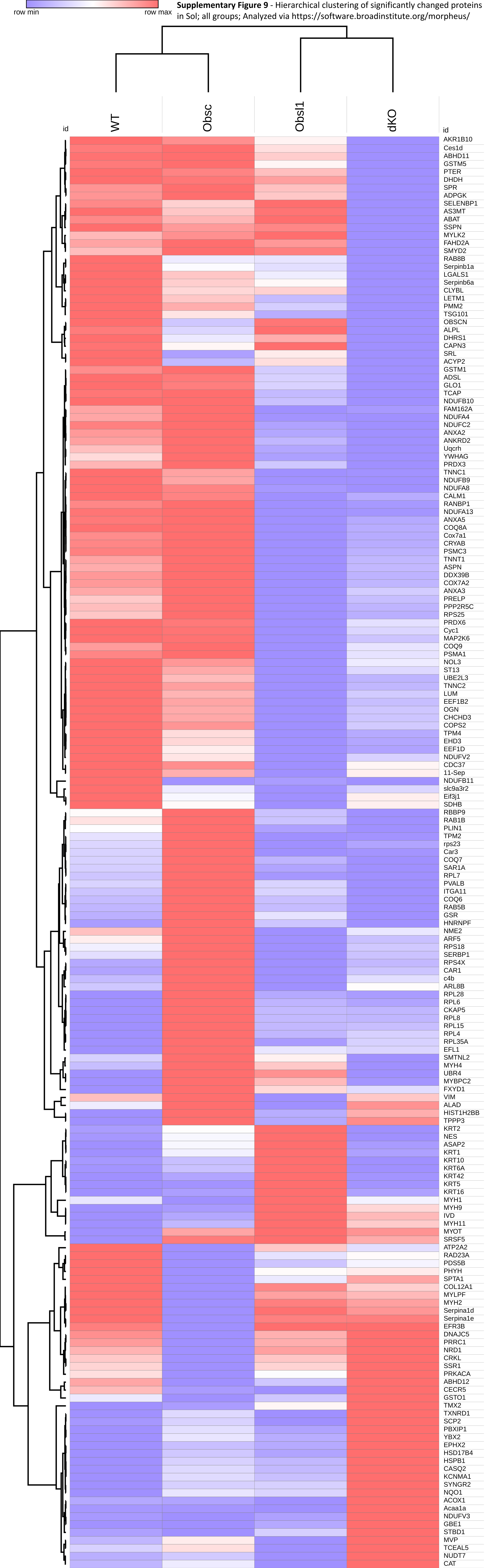

Supplementary Figure 9. Hierarchical clustering of significantly altered proteins identified in soleus (Sol) muscles of control (CTL), obscurin knockout (Obsc), skeletal muscle specific Obsl1 knockout (Obsl1) and double knockout (dKO) mice using Morpheus. Data from this analysis has been used to generate Figure 4a.

Supplementary Table 1. Oligonucleotides used for this study.

**name**	***sequence***	***notes***
Obsl1_LoxP1.fwd	GGCAGCTGCAGGCGACAAAGTC	Oligonucleotides used for genotyping. LoxP site located 5' of coding exon 1; expected sizes: wildtype locus 528bp; floxed locus ~570bp
Obsl1_LoxP1.rev	CCGCCTTTCTCCCACACGACAG	
Obsl1_LoxP2.fwd	GGTTCTGCTCACCTTGG	Oligonucleotides used for genotyping. LoxP site located 3' of coding exon 4; expected sizes: wildtype locus 518bp; floxed locus ~560bp
Obsl1_LoxP2.rev	GGAACTAAACCACTAACCAACGAGG	
Obsl1_wt.fwd	GATTTAAAGCAGGAGTGTTGATGG	Oligonucleotides used for genotyping. amplifies genomic DNA spanning a fragment from intron 3-4 to exon 4; expected size of wildtype locus: 315bp
Obsl1_wt.rev	TGTGCAGATGCGGAAGTGATAGTC	
Obsl1_southern_probe.fwd	GTCCCACCCTAGAGACCAT	generation of probe for southern blot analysis; expected band size of the probe: 561bp; genomic DNA digested with NsiI
Obsl1_southern_probe.rev	GGTGATGGTGAAGGAAGCCGACTC	

Supplementary Table 2. Primary antibodies used for this study.

**target**	***source***	***notes***
sarcomeric alpha actinin-2 (ACTN2)	clone EA-53; Mob 227-05; Diagnostic Biosystems	
pan sarcomeric myosin (heavy chain)	deposited by Blau, H.M. clone A4.1025; DSHB	
Obsl1	kind gift of Dr. Mathias Gautel; King's College London	epitope in Ig-domain 1; knockout validated
Obsl1	ab204075; abcam	epitope targeting aa 1267-1355 (Ig domain 14); knockout validated
Obscurin	generated in the Chen and Lange laboratories	epitope located within region covering IQ-Ig64; knockout validated
myomesin-1	kind gift of Dr. Agarkova; ETH Zurich	
myomesin-2 (M-protein)	clone AA259; kind gift of Dr. Ehler; Kings College London developed by Dr. Fürst; University of Bonn	
Dystrophin	deposited by Morris, G.E.; clone MANDRA1 (7A10); DSHB	
Utrophin	deposited by Morris, G.E.; clone MANCHO14(4E1); DSHB	
beta-dystroglycan	deposited by Morris, G.E. clone MANDAG2(7D11); DSHB	
alpha-dystrobrevin	sc-365102; SCBT	
alpha-tubulin	deposited by Walsh, C. clone AA4.3; DSHB	
beta-tubulin	deposited by Klymkowsky, M. clone E7; DSHB	
beta-1D integrin	kind gift of Dr. Robert Ross; UC San Diego	
alpha-syntrophin	sc-13757; SCBT	
TRCP1	ACC-010; Alome	
caveolin-1	3267; cell signaling	
DHPR alpha-2 subunit	ab2864; abcam	

**target**	***source***	***notes***
nNOS	MAB2416-SP; NovusBio	
Dysferlin	ab124684; abcam	
Filamin-C	NBP-189300; NovusBio	
alpha-B crystallin	ADI-SPA-223; Enzo	
sAnk1.5	generated in the Sorrentino laboratory	knockout validated
sAnk1.5	ARP42566_T100; Aviva Sysbio	
Serca1	12293; cell signaling	
RyR	clone 34C; ALX-804-016-R100; Enzo	
Sarcalumenin	developed by K. P. Campbell<br>clone XIIC4; DSHB	
Calsequestrin 2	sc-16576; SCBT	
Calsequestrin 1	sc-28274; SCBT	
Oxphos antibody cocktail	45-8099; Novex; Life Technologies	
Uqcrb	ab190360; abcam	
Prdx3	ab673349; abcam	
GFP	11814460001; Roche	
Xin1-alpha	sc-68409; SCBT	
tropomyosin 1 (TPM1)	ab133292; abcam	
titin M8 epitope	kind gift of Dr. Mathias Gautel; King’s College London	
beta-actin	sc-47778; SCBT	
