# Supplementary material for: Murine obscurin and Obsl1 have functionally redundant roles in sarcolemmal integrity, sarcoplasmic reticulum organization, and muscle metabolism
Source: Commun Biol. 2019 May 9;2:178. doi: 10.1038/s42003-019-0405-7 (PMC6509138; doi:10.1038/s42003-019-0405-7)
Supplement: Supplementary file 2 — Description of Additional Supplementary Files [file 42003_2019_405_MOESM2_ESM.docx]

**Description of Additional Supplementary Files**

File Name: Supplementary Data 1

Description: Proteome data. Significantly altered proteins identified by

proteome analysis of tibialis anterior (TA) and soleus (Sol) muscles. This file contains raw

values and calculated data for protein fold changes, including their significance for the

shown volcano plots, comparisons between altered proteins identified in TA vs. Sol

muscles, as well as summary data for the enrichment and pathway analyses.

Sheet: TA_proteins – Significantly altered proteins identified in TA

Sheet: Sol_proteins – Significantly altered proteins in Sol

Sheet: Comparison TASol – Comparison between proteins altered in TA & Sol

(data used to generate Figure 4b)

Sheet: volcano plot TA WT-dKO – data used to generate volcano plots for

proteins deregulated in TA

Sheet: volcano plot Sol WT-dKO – data used to generate volcano plots for

proteins deregulated in Sol

Sheet: Enrichment analysis TA – enrichment analysis for proteins deregulated in

TA (Wt-dKO)

Sheet: Enrichment analysis Sol – enrichment analysis for proteins deregulated in

Sol (Wt-dKO)

File Name: Supplementary Data 2

Description: Raw output from metascape pathway and enrichment analyses of proteome datasets. Metascape enrichment analysis pathways Sol+TA all groups sign changed proteins

File Name: Supplementary Data 3

Description: Raw output from metascape pathway and enrichment analyses of proteome datasets. Metascape enrichment analysis pathways TA all groups sign changed proteins

File Name: Supplementary Data 4

Description: Raw output from metascape pathway and enrichment analyses of proteome datasets. Metascape enrichment analysis pathways TA all groups sign changed proteins

File Name: Supplementary Data 5

Description: Comparison between proteome data versus putative and verified Obsl1 interaction partners, or transcriptome data from 3M patient fibroblasts.

File Name: Supplementary Data 6

Description: Uncropped blot images for all immunoblot data presented in the manuscript.
