# Supplementary material for: Murine obscurin and Obsl1 have functionally redundant roles in sarcolemmal integrity, sarcoplasmic reticulum organization, and muscle metabolism
Source: Commun Biol. 2019 May 9;2:178. doi: 10.1038/s42003-019-0405-7 (PMC6509138; doi:10.1038/s42003-019-0405-7)
Supplement: Supplementary file 3 — Reporting Summary [file 42003_2019_405_MOESM3_ESM.pdf]

nature research

Corresponding author(s): Stephan Lange

Last updated by author(s): Mar 8, 2019

Reporting Summary

Nature Research wishes to improve the reproducibility of the work that we publish. This form provides structure for consistency and transparency in reporting. For further information on Nature Research policies, see Authors & Referees and the Editorial Policy Checklist.

Statistics

For all statistical analyses, confirm that the following items are present in the figure legend, table legend, main text, or Methods section.

n/a	Confirmed	
☐	☒	The exact sample size (*n*) for each experimental group/condition, given as a discrete number and unit of measurement
☐	☒	A statement on whether measurements were taken from distinct samples or whether the same sample was measured repeatedly
☐	☒	The statistical test(s) used AND whether they are one- or two-sided<br>*Only common tests should be described solely by name; describe more complex techniques in the Methods section.*
☒	☐	A description of all covariates tested
☐	☒	A description of any assumptions or corrections, such as tests of normality and adjustment for multiple comparisons
☐	☒	A full description of the statistical parameters including central tendency (e.g. means) or other basic estimates (e.g. regression coefficient) AND variation (e.g. standard deviation) or associated estimates of uncertainty (e.g. confidence intervals)
☐	☒	For null hypothesis testing, the test statistic (e.g. *F*, *t*, *r*) with confidence intervals, effect sizes, degrees of freedom and *P* value noted<br>*Give P values as exact values whenever suitable.*
☒	☐	For Bayesian analysis, information on the choice of priors and Markov chain Monte Carlo settings
☒	☐	For hierarchical and complex designs, identification of the appropriate level for tests and full reporting of outcomes
☒	☐	Estimates of effect sizes (e.g. Cohen's *d*, Pearson's *r*), indicating how they were calculated

Our web collection on statistics for biologists contains articles on many of the points above.

Software and code

Policy information about availability of computer code

Data collection	software names and version included in materials & methods
Data analysis	software names and version included in materials & methods

For manuscripts utilizing custom algorithms or software that are central to the research but not yet described in published literature, software must be made available to editors/reviewers. We strongly encourage code deposition in a community repository (e.g. GitHub). See the Nature Research guidelines for submitting code & software for further information.

Data

Policy information about availability of data

All manuscripts must include a data availability statement. This statement should provide the following information, where applicable:

- Accession codes, unique identifiers, or web links for publicly available datasets
- A list of figures that have associated raw data
- A description of any restrictions on data availability

All raw data and accession codes associated with the manuscript are in the manuscript and supplemental information, or will be made available upon request.

Field-specific reporting

Please select the one below that is the best fit for your research. If you are not sure, read the appropriate sections before making your selection.

☒ Life sciences ☐ Behavioural & social sciences ☐ Ecological, evolutionary & environmental sciences

For a reference copy of the document with all sections, see nature.com/documents/nr-reporting-summary-flat.pdf

Life sciences study design

All studies must disclose on these points even when the disclosure is negative.

Sample size	Sample sizes are stated for each figure in the figure or figure legend. If not stated otherwise, biological replicates were used (e.g. for immunoblots, protein level quantifications).
Data exclusions	No data were excluded in the generation of figures/tables, with the exception of two lanes in Figure S7b (upper panel) that did not pass the quality control as judged by ponceau stain (samples ran strange due to malfunction in the gel).
Replication	We used biological replicates to showcase biological variability and reproducibility of findings. Whenever appropriate, different methods were used to verify a finding (e.g. interaction found in yeast 2 hybrid was verified by co-immunoprecipitation).
Randomization	Randomization was done for sex as a biological variable.
Blinding	Some analyses were done in blinded fashion. These include results for figures 2 and any data obtained by the proteome analysis. Specifically, data analysis regarding cross-sectional area measurements were done not by the person who obtained the images. For the proteome analyses, data were generated by the core and core personnel, who had no bias towards the outcome of the experiment.<br>Blinding was done by assigning codes to the samples/sample images before subsequent raw data analyses. Results were later decoded to generate figures and reveal experimental outcomes.

Reporting for specific materials, systems and methods

We require information from authors about some types of materials, experimental systems and methods used in many studies. Here, indicate whether each material, system or method listed is relevant to your study. If you are not sure if a list item applies to your research, read the appropriate section before selecting a response.

Materials & experimental systems

n/a	Involved in the study	
☐	☒	Antibodies
☐	☒	Eukaryotic cell lines
☒	☐	Palaeontology
☐	☒	Animals and other organisms
☒	☐	Human research participants
☒	☐	Clinical data

Methods

n/a	Involved in the study	
☒	☐	ChIP-seq
☒	☐	Flow cytometry
☒	☐	MRI-based neuroimaging

Antibodies

Antibodies used	The full list of antibodies is described in a supplemental table.
Validation	Comments on the validation of antibodies are available in the manuscript and the supplemental table (included whether antibodies were validated with the knockout). In addition, all immunoblot bands in the figrues are depicted with molecular weights as recorded in the raw data.

Eukaryotic cell lines

Policy information about cell lines

Cell line source(s)	Cos1 cells were used in this study to express proteins used in CoIP/Pulldown experiments. Cos1 cells were sourced from ATCC.
Authentication	Authentication of the cell line was done by the supplier (ATCC). We checked if the cell line characteristics (morphology) matched with our observations.
Mycoplasma contamination	Cell line was not tested for presence of mycoplasma.
Commonly misidentified lines<br>(See ICLAC register)	N/A

Animals and other organisms

Policy information about studies involving animals; ARRIVE guidelines recommended for reporting animal research

Laboratory animals	This study involved genetically modified mice. Background strain: black swiss. Age: 3-12 months. Sex: both sexes were used.

Wild animals	N/A
Field-collected samples	N/A
Ethics oversight	All procedures involving animals have been approved by the UC San Diego institutional oversight committee IACUC (protocol No. S13009)

Note that full information on the approval of the study protocol must also be provided in the manuscript.
