# Supplementary material for: Murine obscurin and Obsl1 have functionally redundant roles in sarcolemmal integrity, sarcoplasmic reticulum organization, and muscle metabolism
Source: Commun Biol. 2019 May 9;2:178. doi: 10.1038/s42003-019-0405-7 (PMC6509138; doi:10.1038/s42003-019-0405-7)
Supplement: Supplementary file 9 — Supplementary Data 6 [file 42003_2019_405_MOESM9_ESM.pdf]

uncropped gel images: Blondelle & Marrocco et al. 2019. Communications Biology

Figure 1

Obscurin

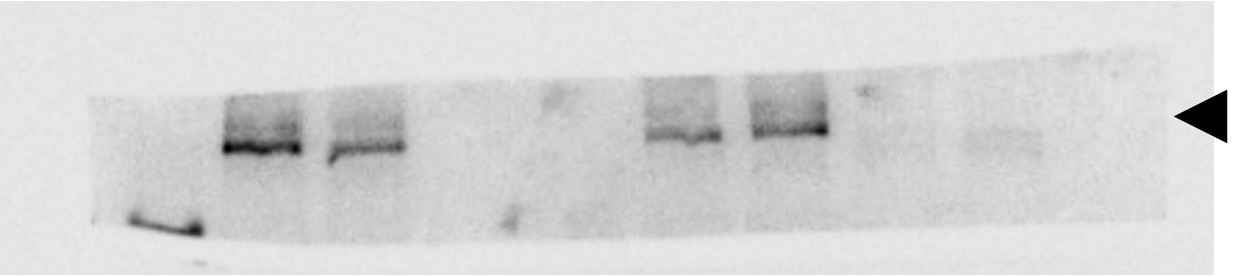

Obsl1 (Ig14)

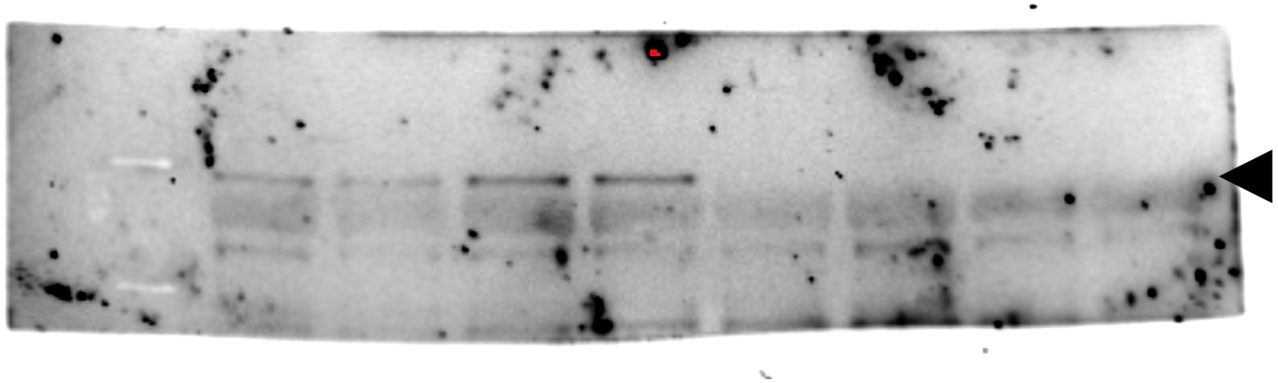

loading: lane1=marker; lanes2-3=controls; lanes3-4=obscurin-ko; lanes 5-6=obsl1-ko; lanes 7-8=dKO

uncropped gel images: Blondelle & Marrocco et al. 2019. Communications Biology

Figure 3a

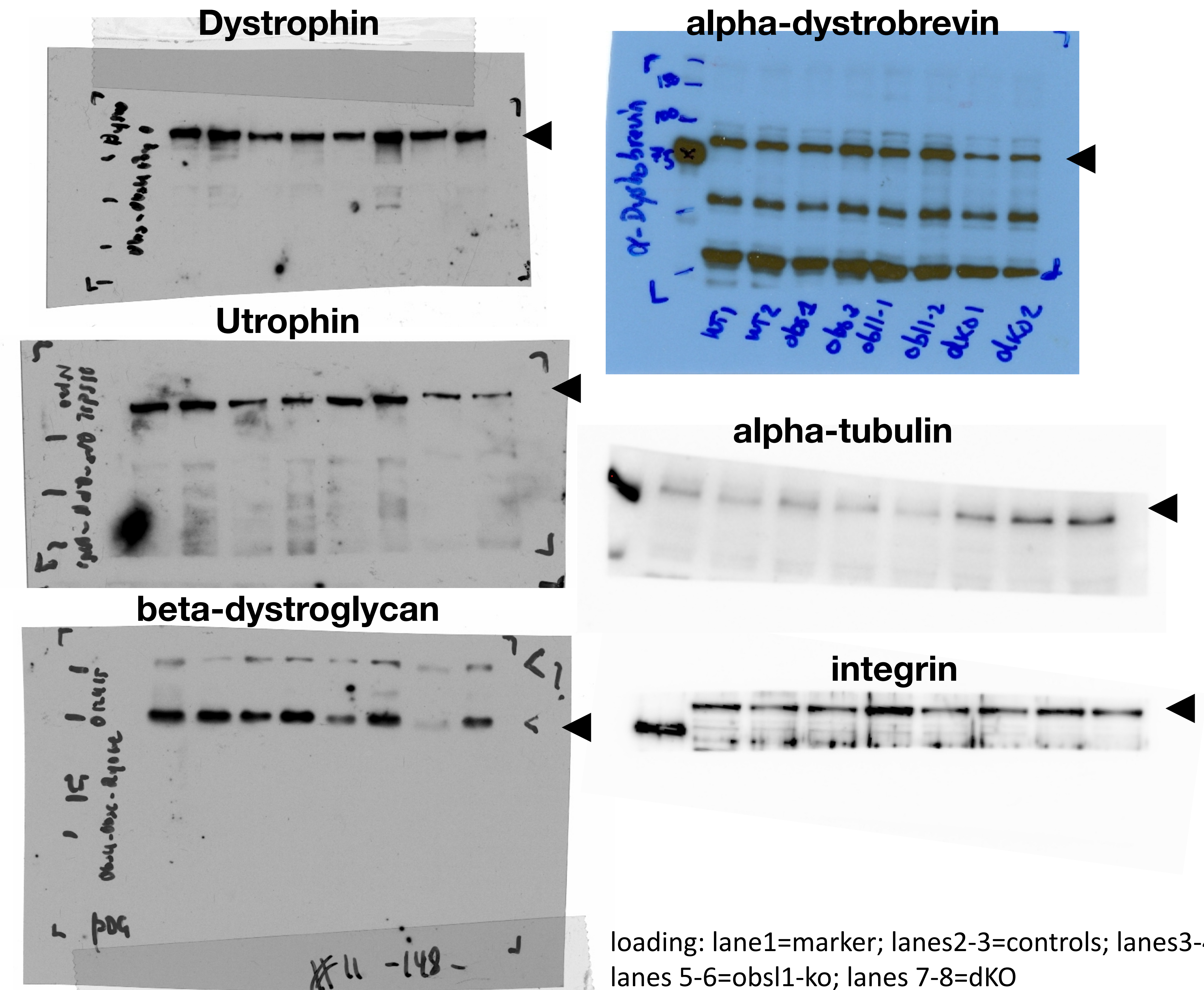

loading: lane1=marker; lanes2-3=controls; lanes3-4=obscurin-ko; lanes 5-6=obsl1-ko; lanes 7-8=dKO

uncropped gel images: Blondelle & Marrocco et al. 2019. Communications Biology

Figure 3d

Dysferlin

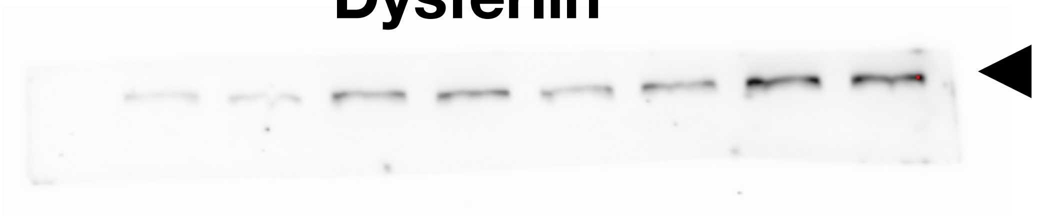

Filamin-C

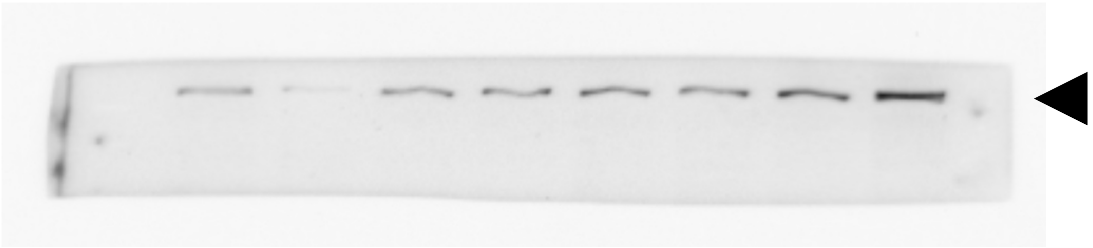

loading: lane1=marker; lanes2-3=controls; lanes3-4=obscurin-ko; lanes 5-6=obsl1-ko; lanes 7-8=dKO

uncropped gel images: Blondelle & Marrocco et al. 2019. Communications Biology

Figure 3e

syntrophin

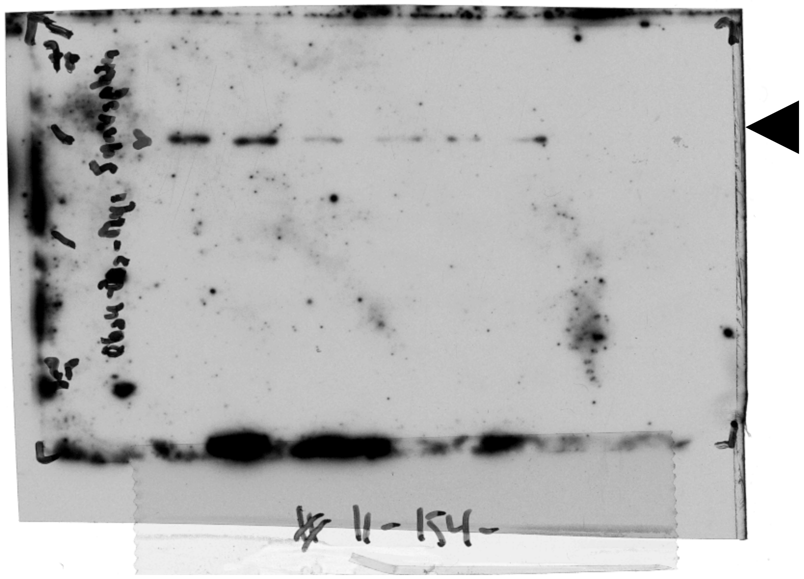

nNOS

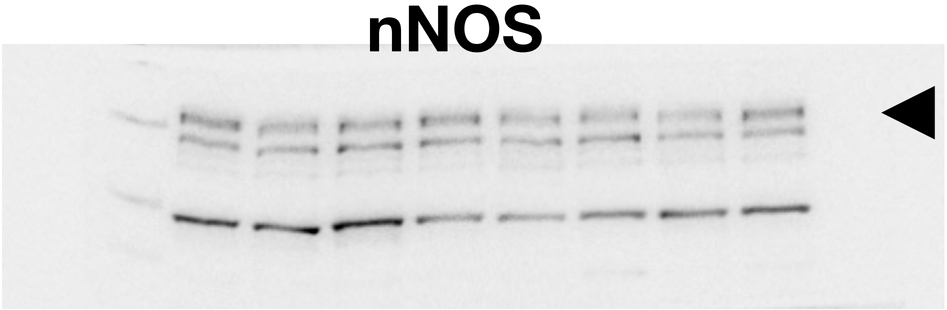

DHPR

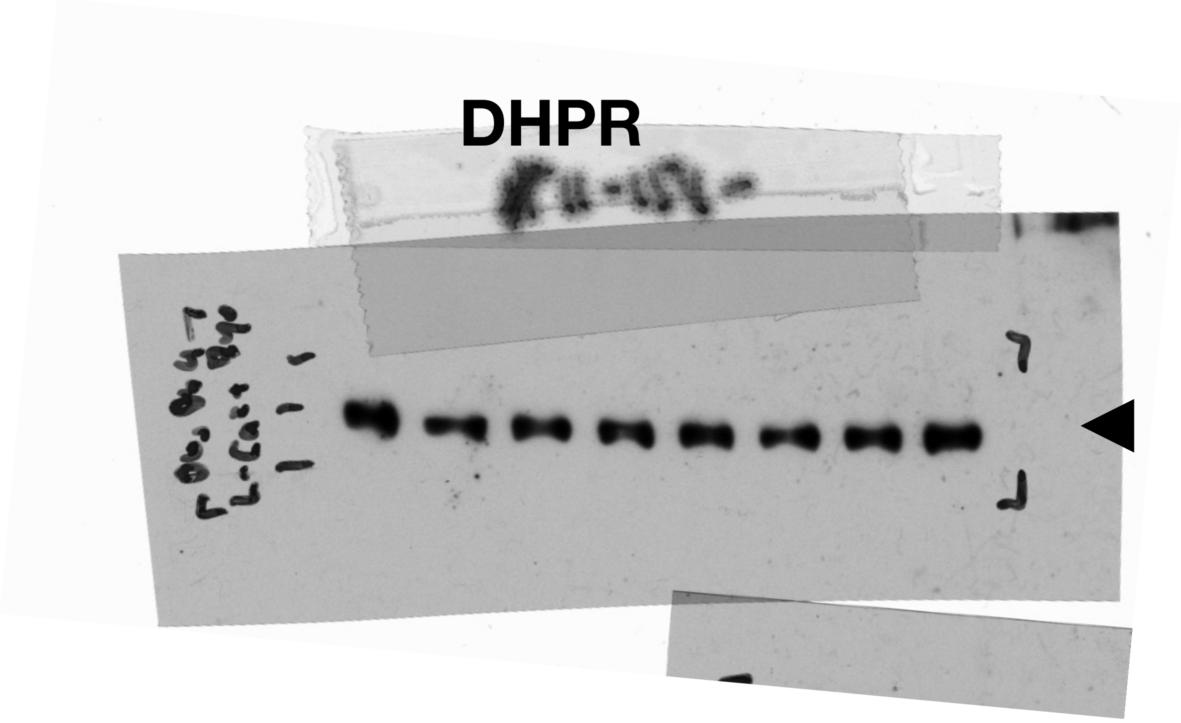

TRPC1

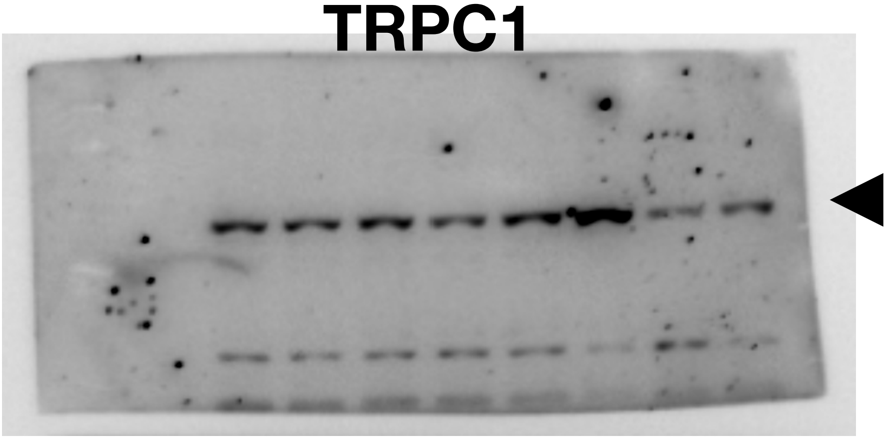

Caveolin-1

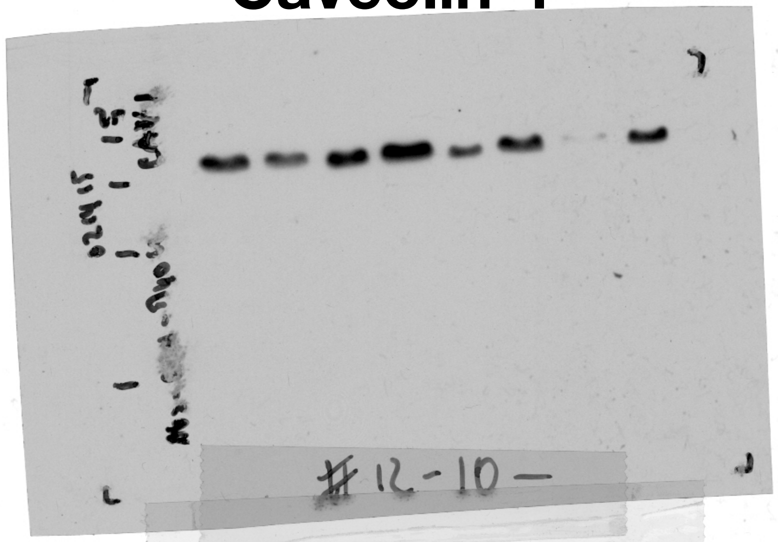

loading: lane1=marker; lanes2-3=controls; lanes3-4=obscurin-ko; lanes 5-6=obsl1-ko; lanes 7-8=dKO

uncropped gel images: Blondelle & Marrocco et al. 2019. Communications Biology

Figure 4

crystallin aB (TA)

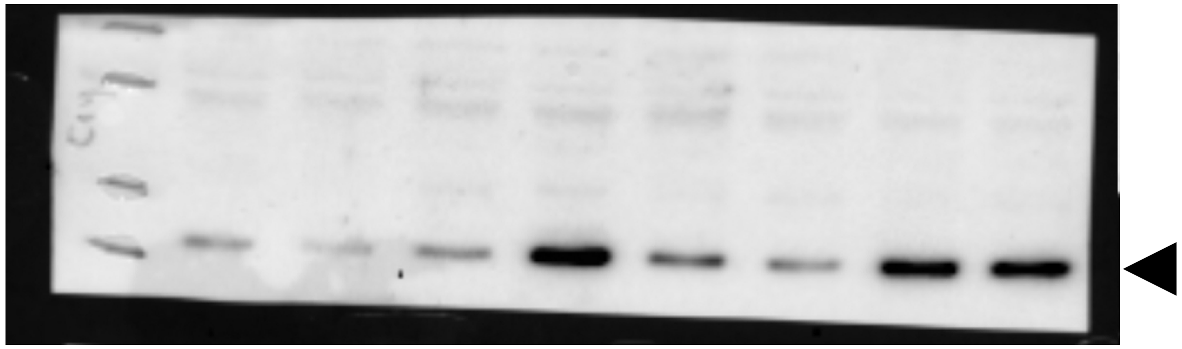

crystallin aB (Sol)

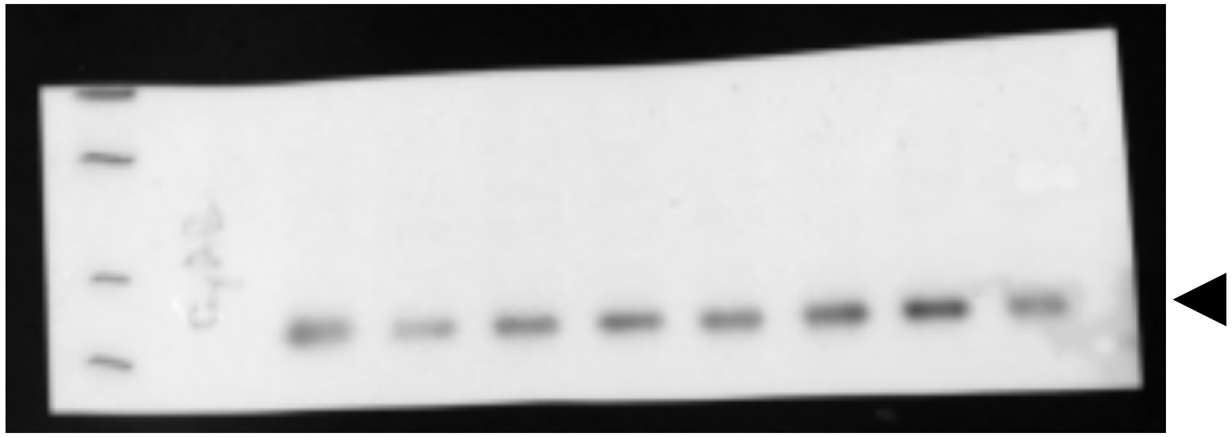

loading: lane1=marker; lanes2-3=controls; lanes3-4=obscurin-ko; lanes 5-6=obsl1-ko; lanes 7-8=dKO

uncropped gel images: Blondelle & Marrocco et al. 2019. Communications Biology

Figure 5b

sAnk1.5

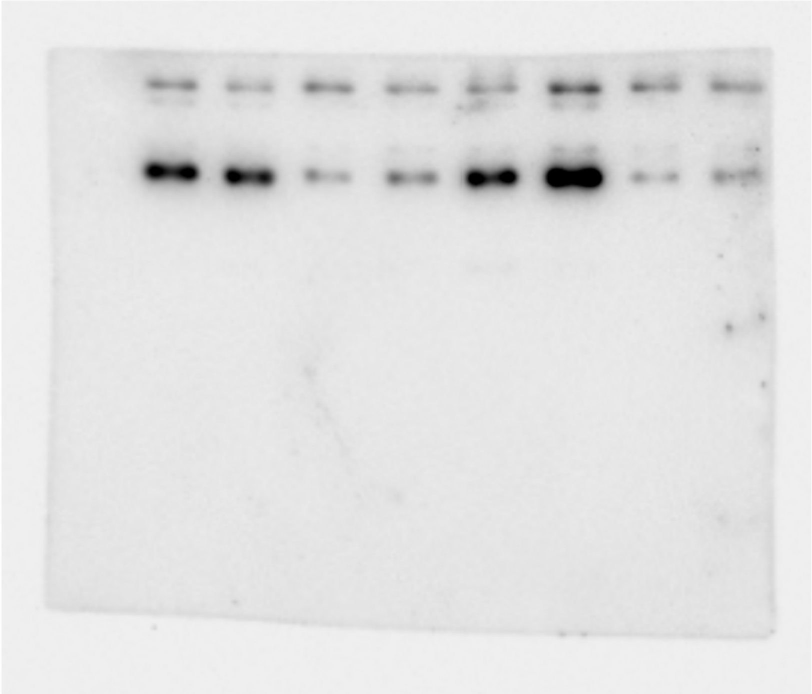

RyR

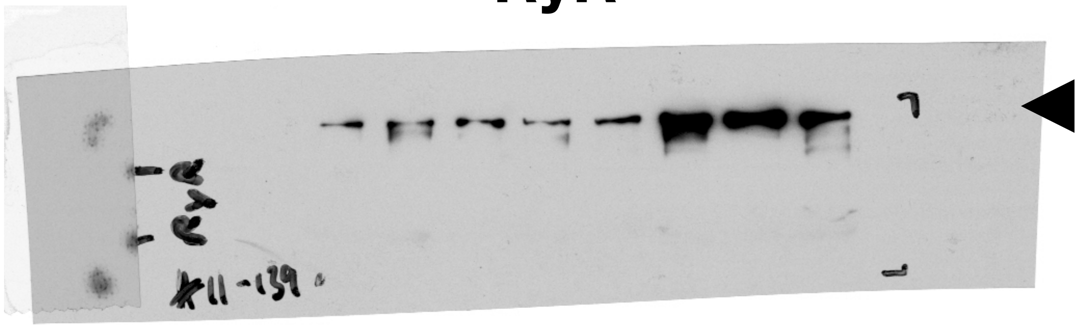

SERCA

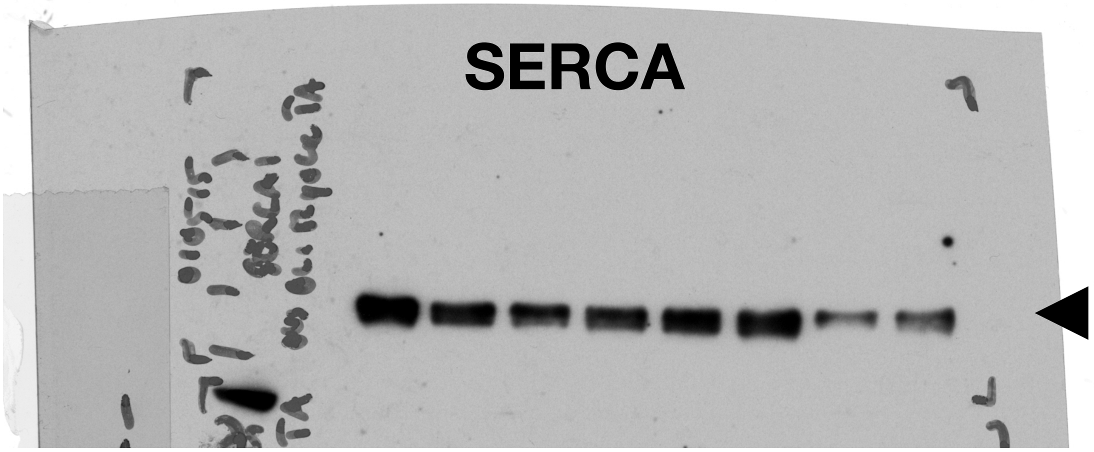

loading: lane1=marker; lanes2-3=controls; lanes3-4=obscurin-ko; lanes 5-6=obsl1-ko; lanes 7-8=dKO

uncropped gel images: Blondelle & Marrocco et al. 2019. Communications Biology

Figure 5c

Sarcalumenin

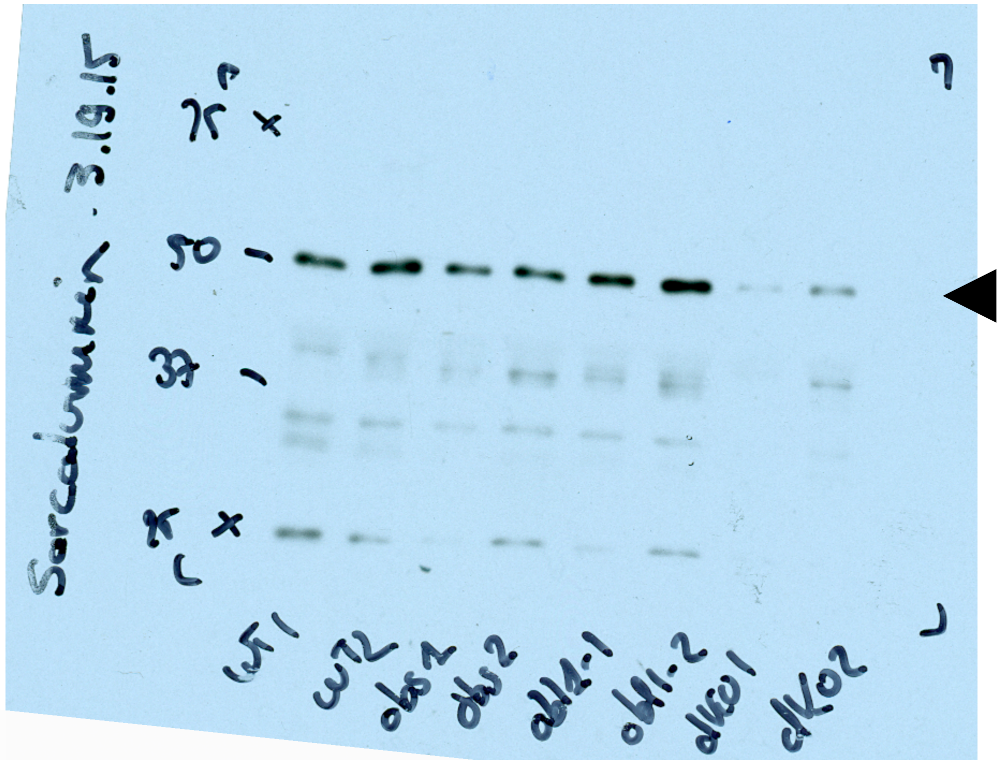

Calseq. 1

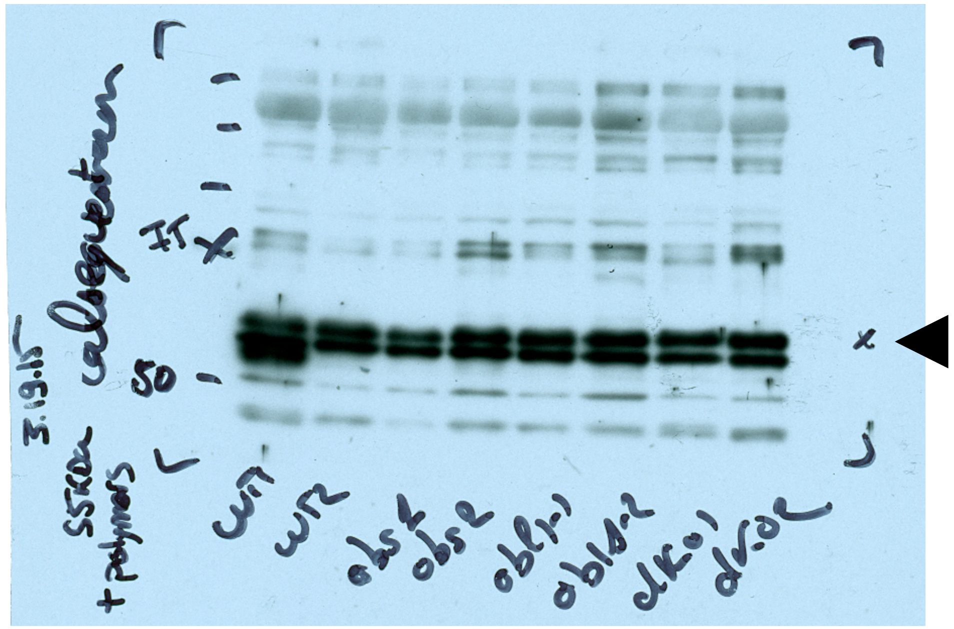

Calseq. 2

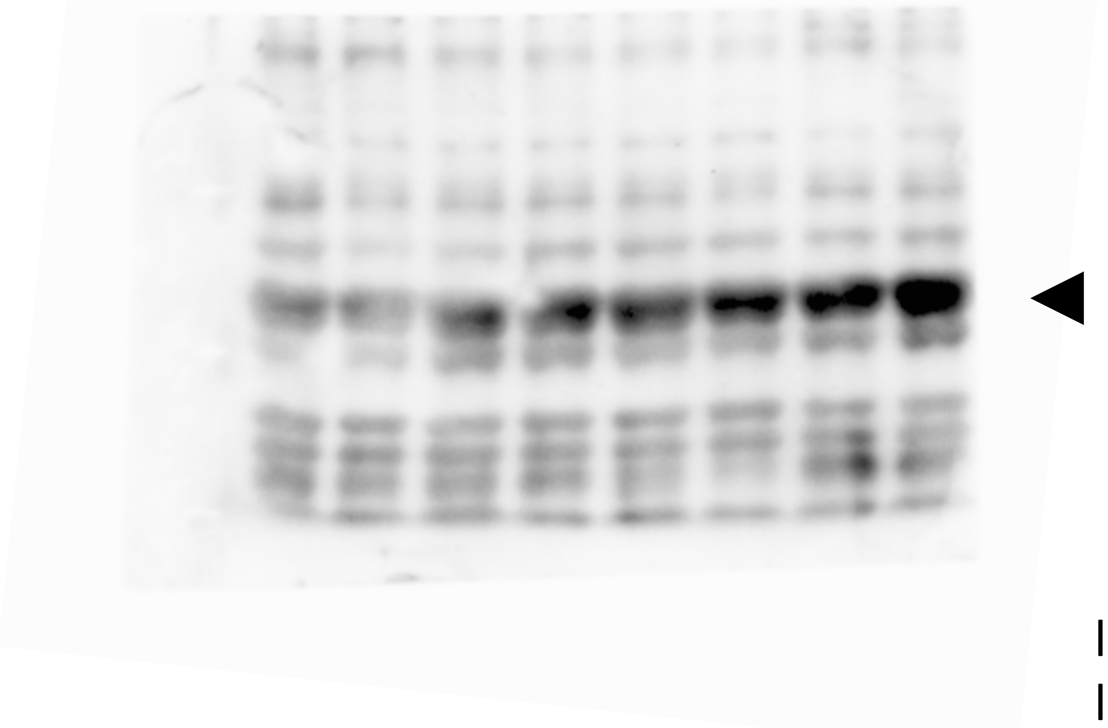

loading: lane1=marker; lanes2-3=controls; lanes3-4=obscurin-ko; lanes 5-6=obsl1-ko; lanes 7-8=dKO

uncropped gel images: Blondelle & Marrocco et al. 2019. Communications Biology

Figure 6c

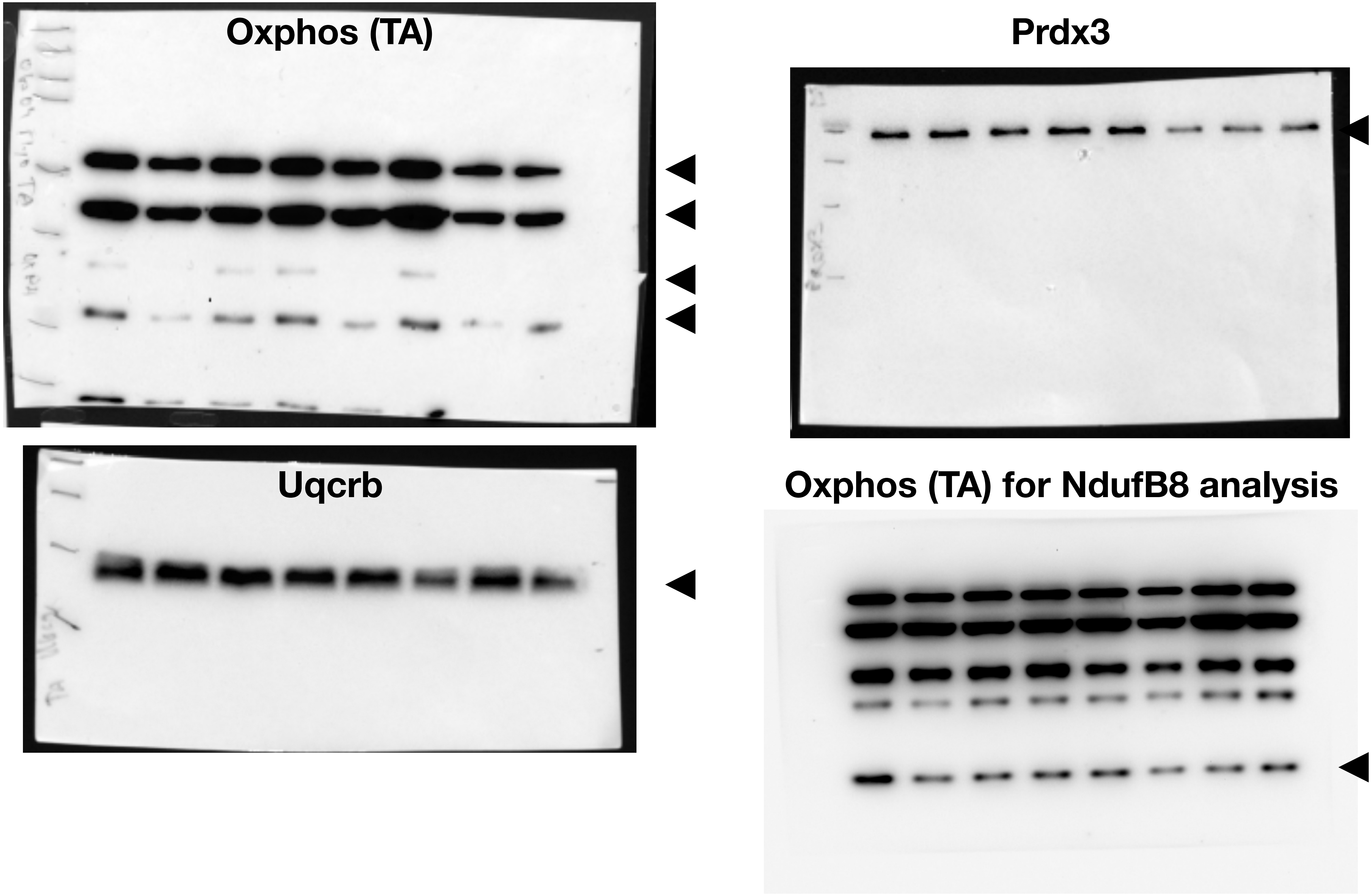

loading: lane1=marker; lanes2-3=controls; lanes3-4=obscurin-ko; lanes 5-6=obsl1-ko; lanes 7-8=dKO

uncropped gel images: Blondelle & Marrocco et al. 2019. Communications Biology

Figure 6c

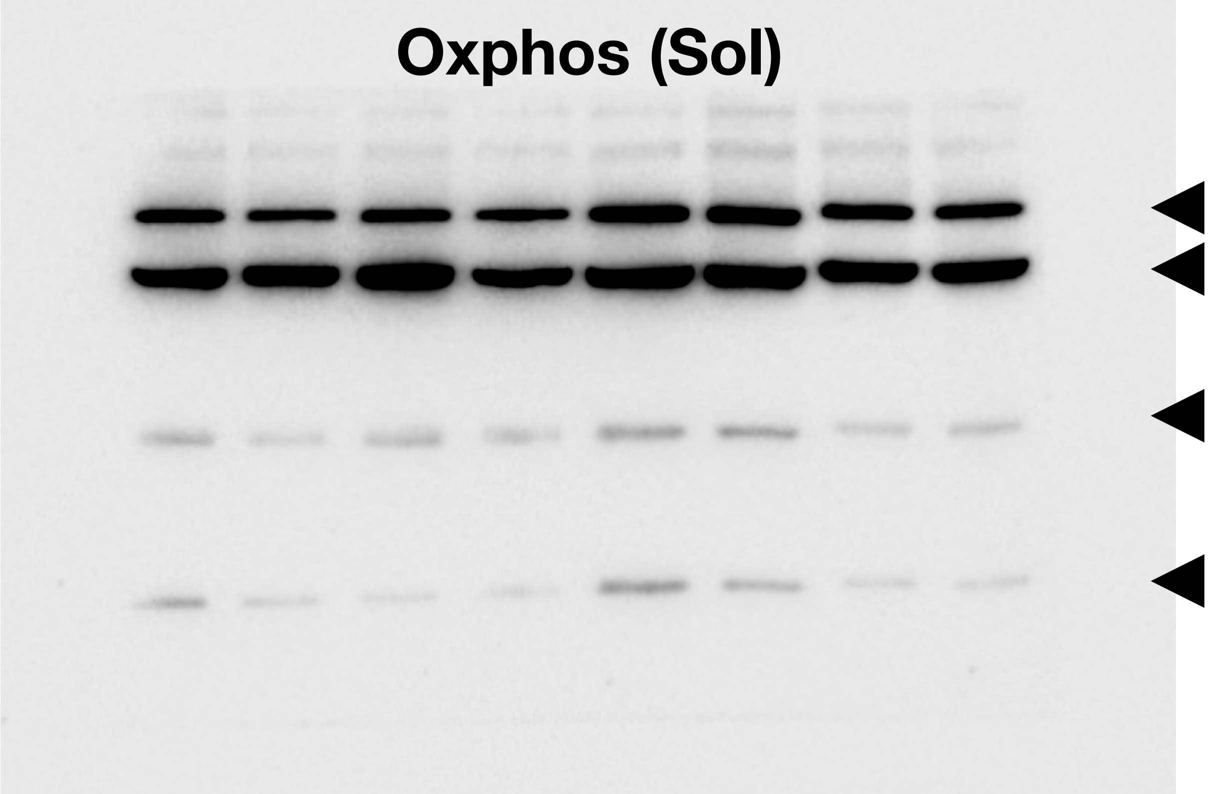

loading: lane1=marker; lanes2-3=controls; lanes3-4=obscurin-ko; lanes 5-6=obsl1-ko; lanes 7-8=dKO

uncropped gel images: Blondelle & Marrocco et al. 2019. Communications Biology

Figure S1b

Obsl1-Ig1

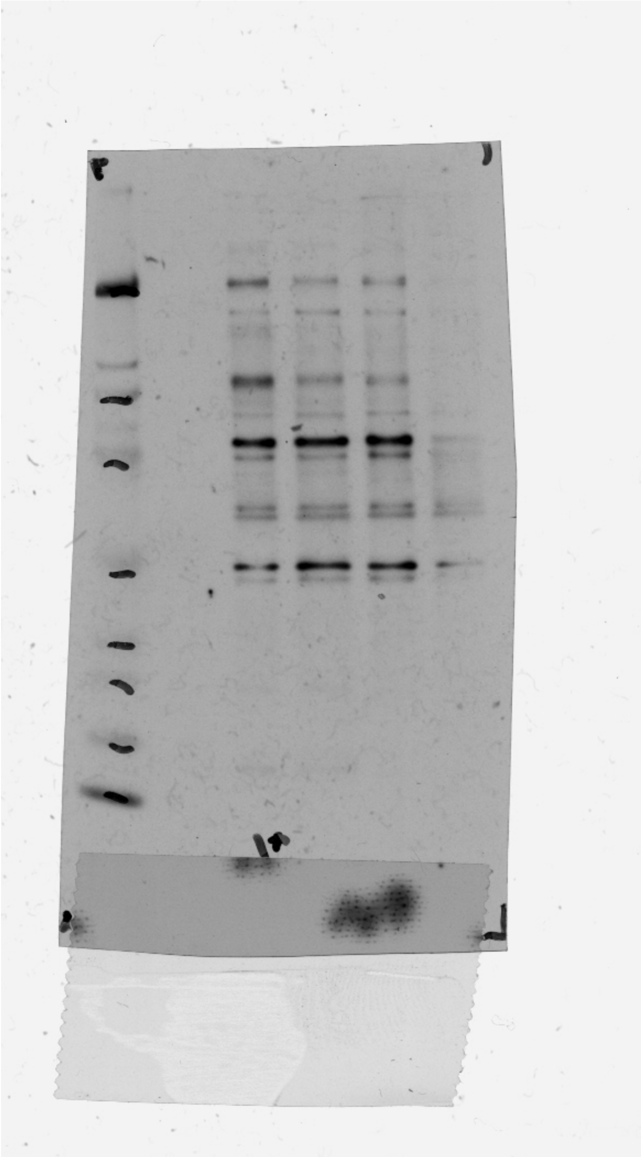

GAPDH

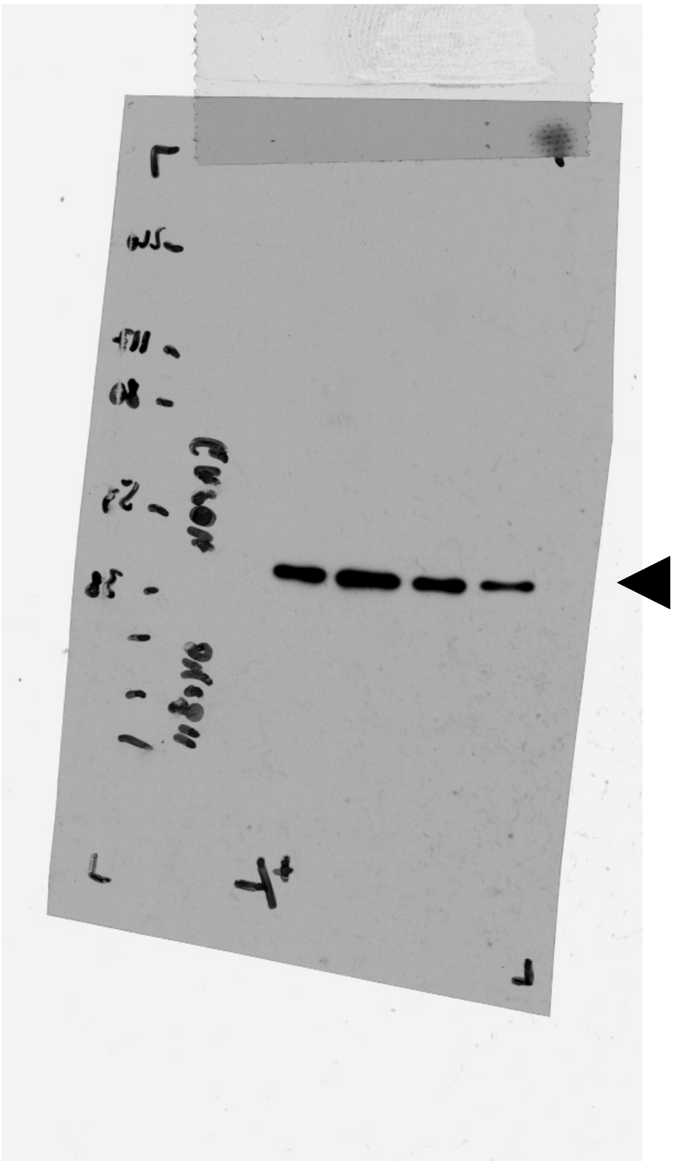

uncropped gel images: Blondelle & Marrocco et al. 2019. Communications Biology

Figure S1c, S1e

Obscurin Iq-64

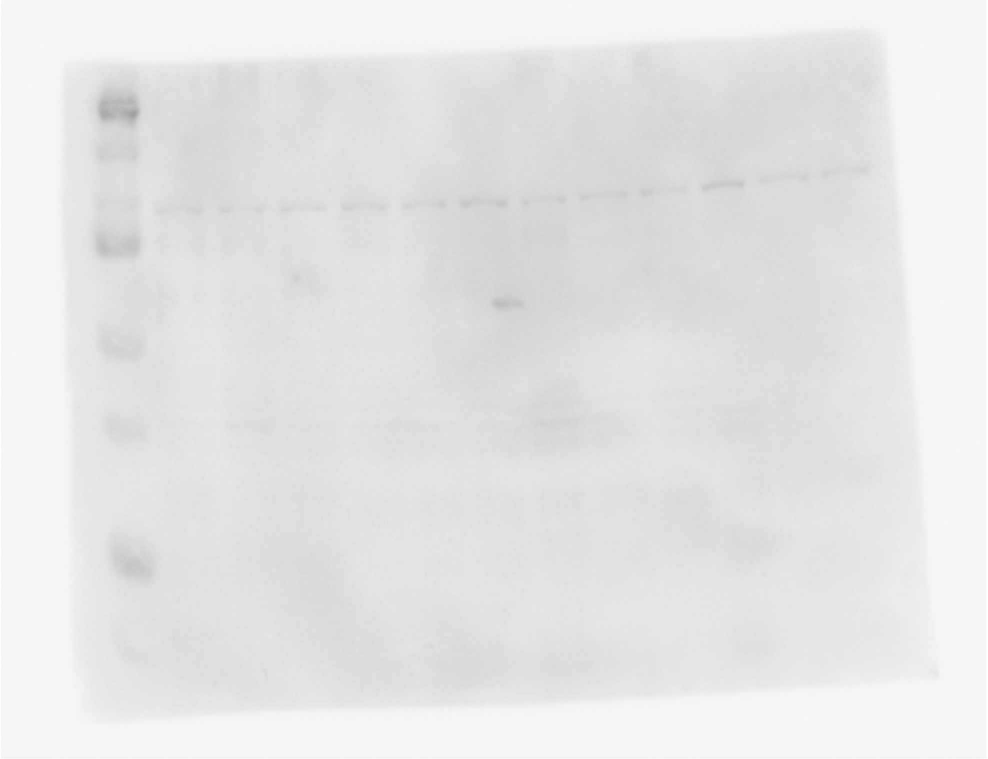

loading: lane1=marker; lanes 2-4=controls; lanes 5-7=obscurin-ko; lanes 8-10=obsl1-ko; lanes 11-13=dKO

myosin

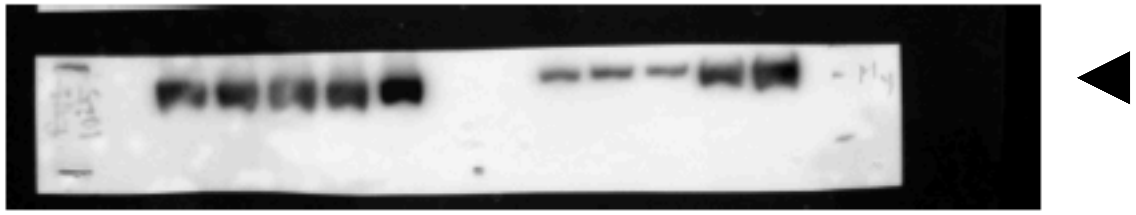

loading: lane1=marker; lanes 3-7=inputs; lanes 10-14=IP;

GFP

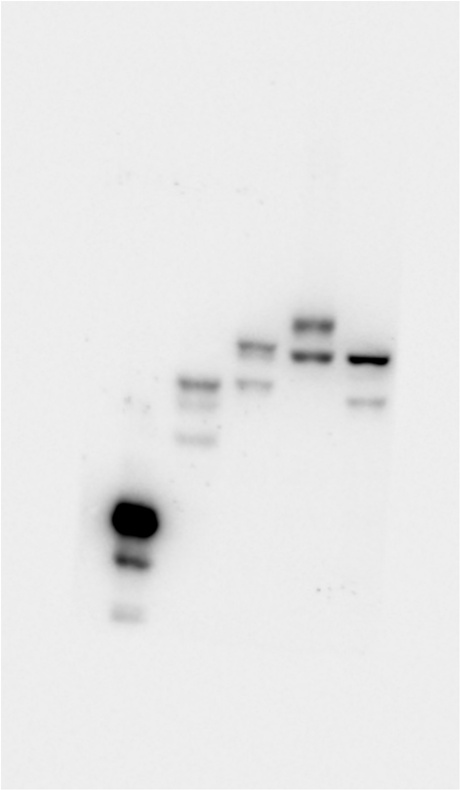

loading: lane1=marker; lanes 3-7=inputs

uncropped gel images: Blondelle & Marrocco et al. 2019. Communications Biology

Figure S1g

myomesin 1

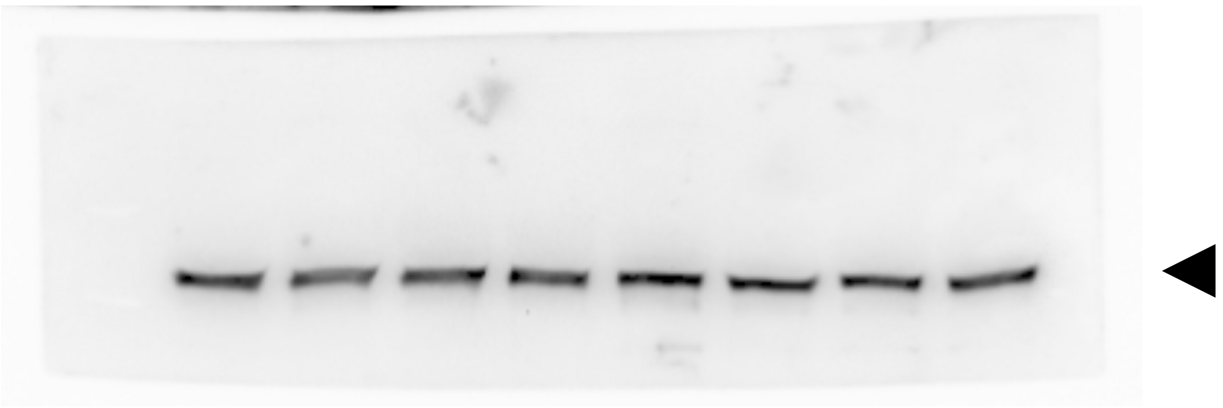

myomesin 2

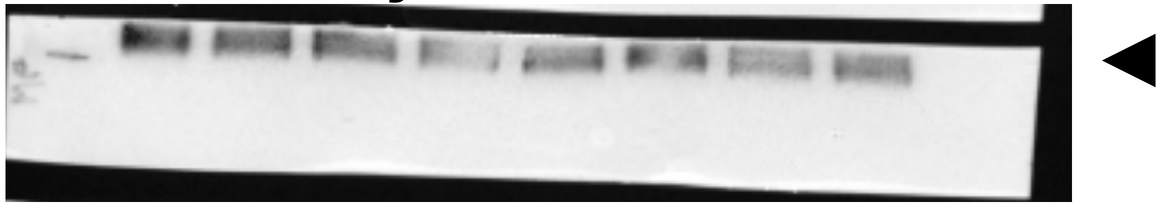

loading: lane 1=marker; lanes 2-3=controls; lanes 3-4=obscurin-ko; lanes 5-6=obsl1-ko; lanes 7-8=dKO

uncropped gel images: Blondelle & Marrocco et al. 2019. Communications Biology

Figure S2a

tubulin

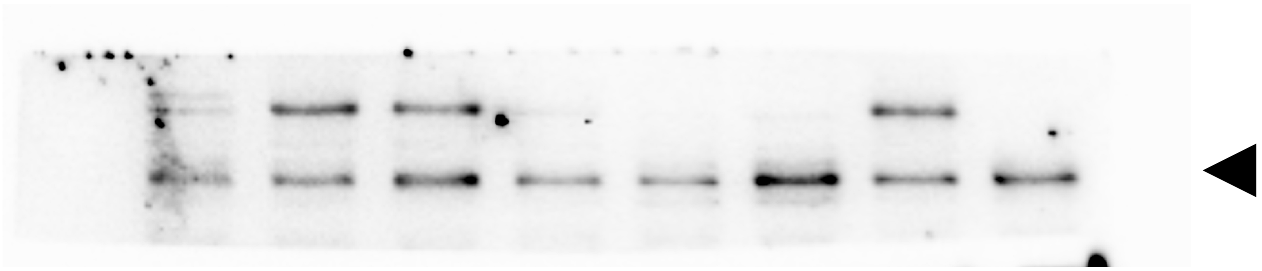

loading: lane 1=marker; lanes 2-3=controls; lanes 3-4=obscurin-ko; lanes 5-6=obsl1-ko; lanes 7-8=dKO

uncropped gel images: Blondelle & Marrocco et al. 2019. Communications Biology

Figure S4a, S4e

GAPDH

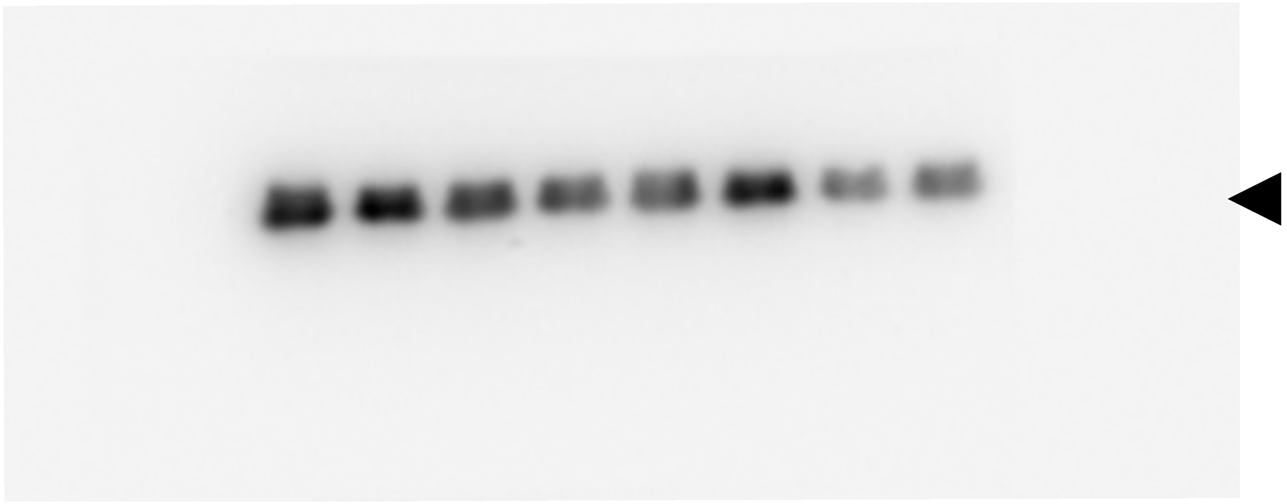

loading: lane 1=marker; lanes 2-3=controls;
lanes 3-4=obscurin-ko; lanes 5-6=obsl1-ko;
lanes 7-8=dKO

Uqcrb

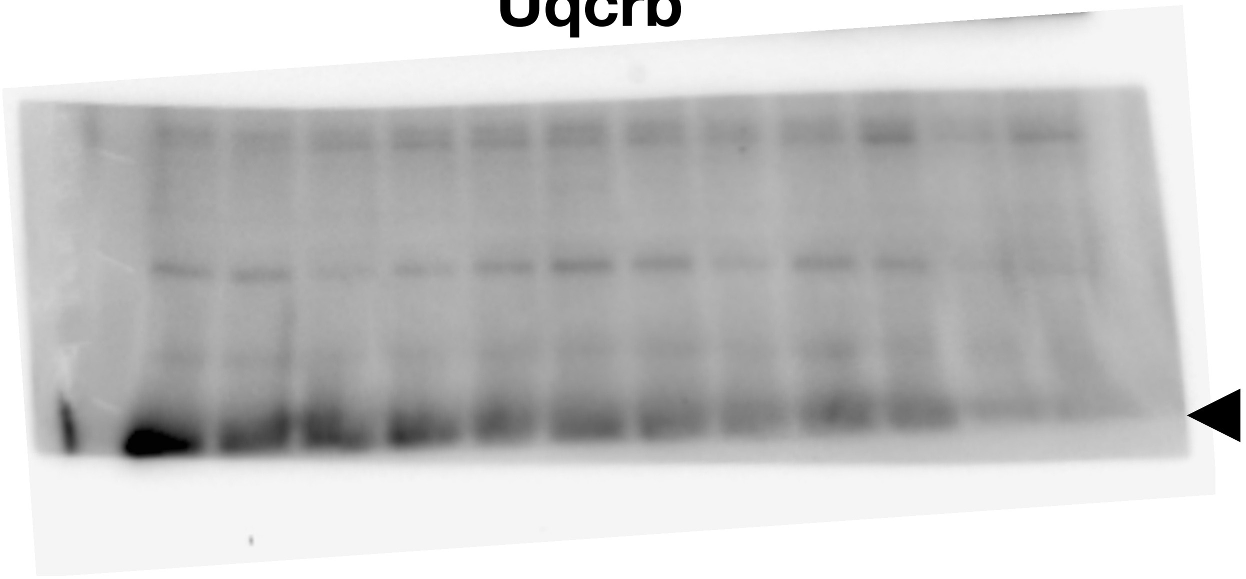

Prdx3

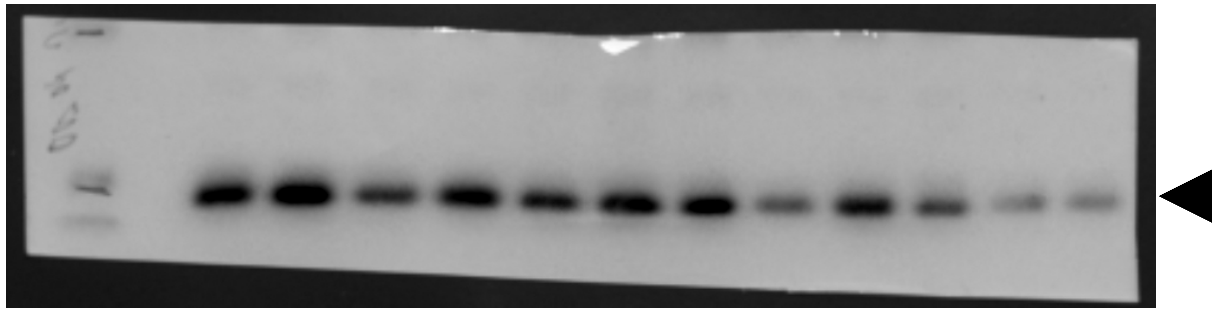

loading: lane1=marker; lanes 2-4=controls;
lanes 5-7=obscurin-ko; lanes 8-10=obsl1-ko;
lanes 11-13=dKO

uncropped gel images: Blondelle & Marrocco et al. 2019. Communications Biology

Figure S7a

dystrophin

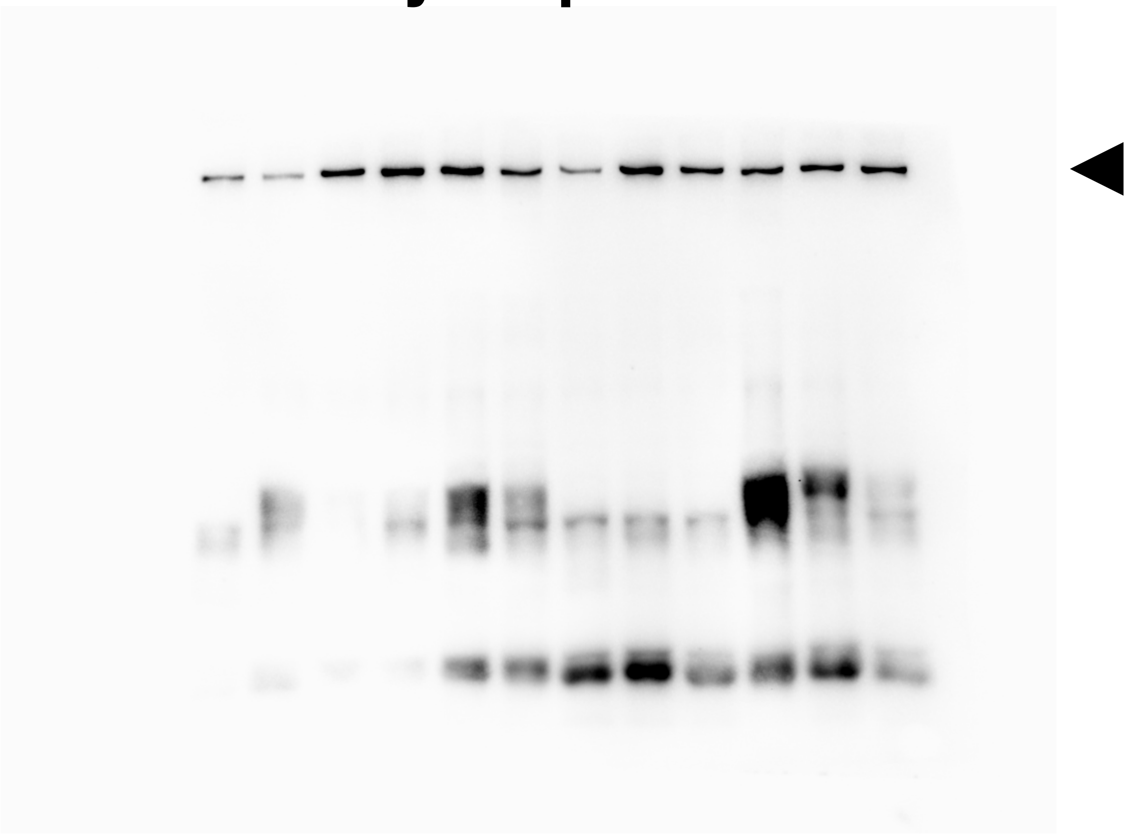

utrophin

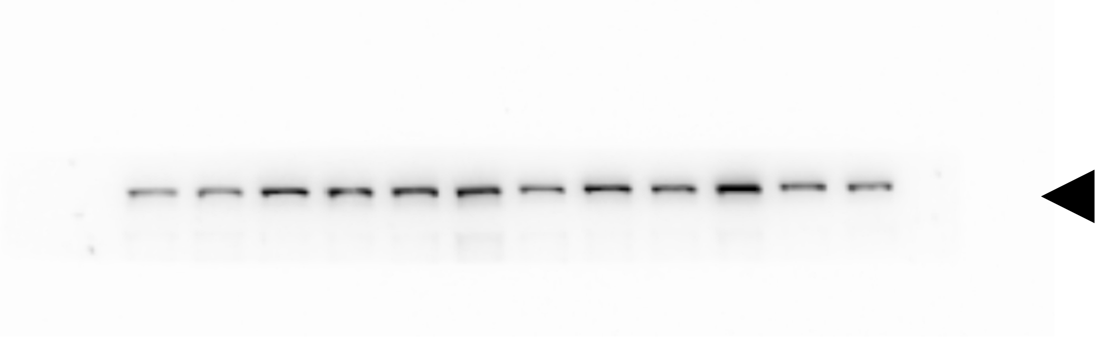

beta-dystroglycan

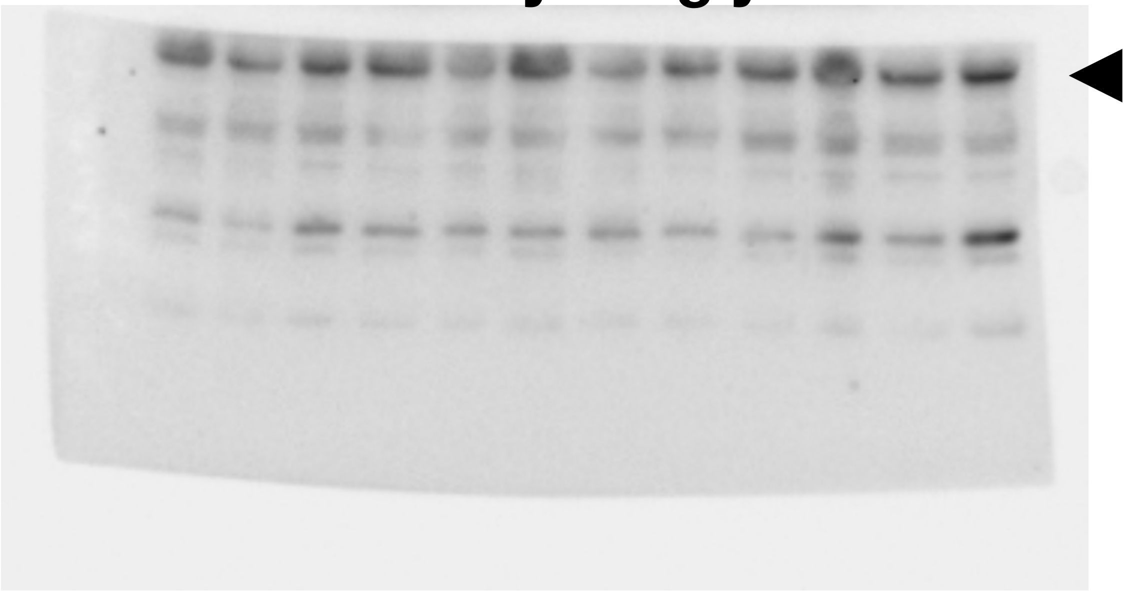

alpha-dystrobrevin

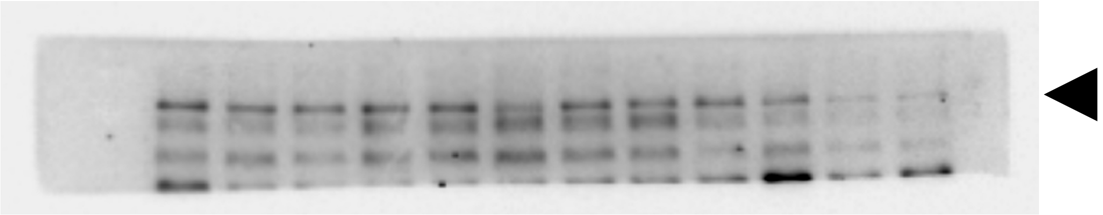

alpha-tubulin

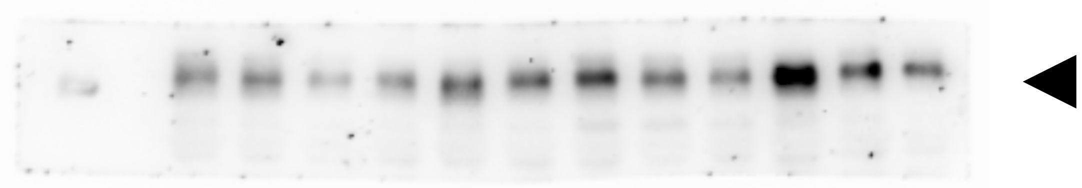

loading: lane1=marker; lanes 2-4=controls; lanes 5-7=obscurin-ko; lanes 8-10=obsl1-ko; lanes 11-13=dKO

uncropped gel images: Blondelle & Marrocco et al. 2019. Communications Biology

Figure S7a

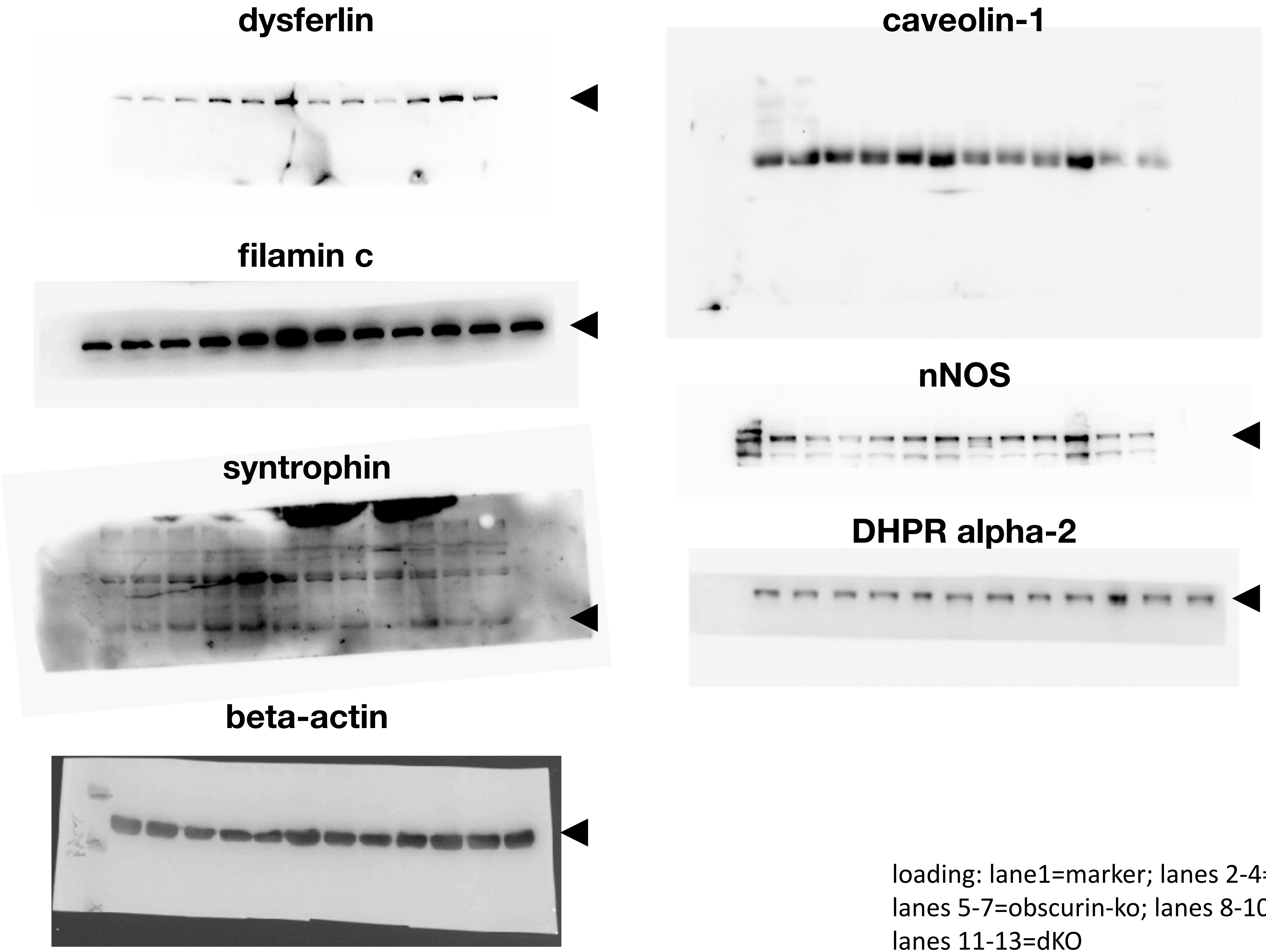

loading: lane1=marker; lanes 2-4=controls; lanes 5-7=obscurin-ko; lanes 8-10=obsl1-ko; lanes 11-13=dKO

uncropped gel images: Blondelle & Marrocco et al. 2019. Communications Biology

Figure S7b

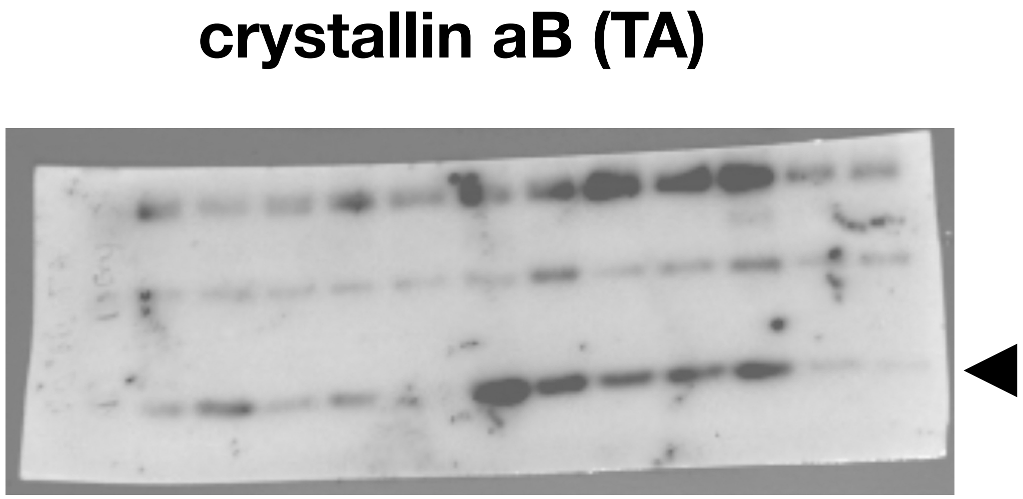

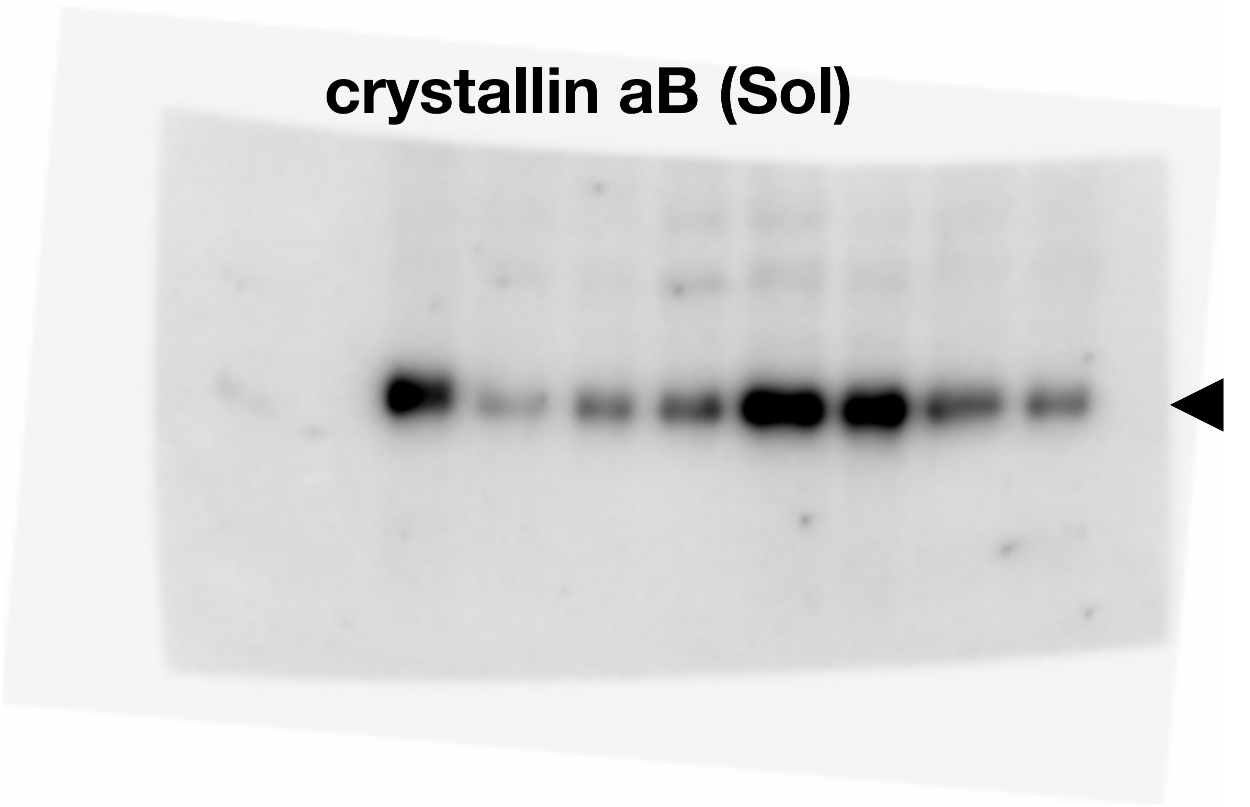

loading: lane1=marker; lanes 2-4=controls; lanes 5-7=obscurin-ko; lanes 8-10=obsl1-ko; lanes 11=dKO; lanes 12-13 - not used for analysis

loading: lane 1=marker; lanes 2-3=controls; lanes 3-4=obscurin-ko; lanes 5-6=obsl1-ko; lanes 7-8=dKO

uncropped gel images: Blondelle & Marrocco et al. 2019. Communications Biology

Figure S7c

RyR

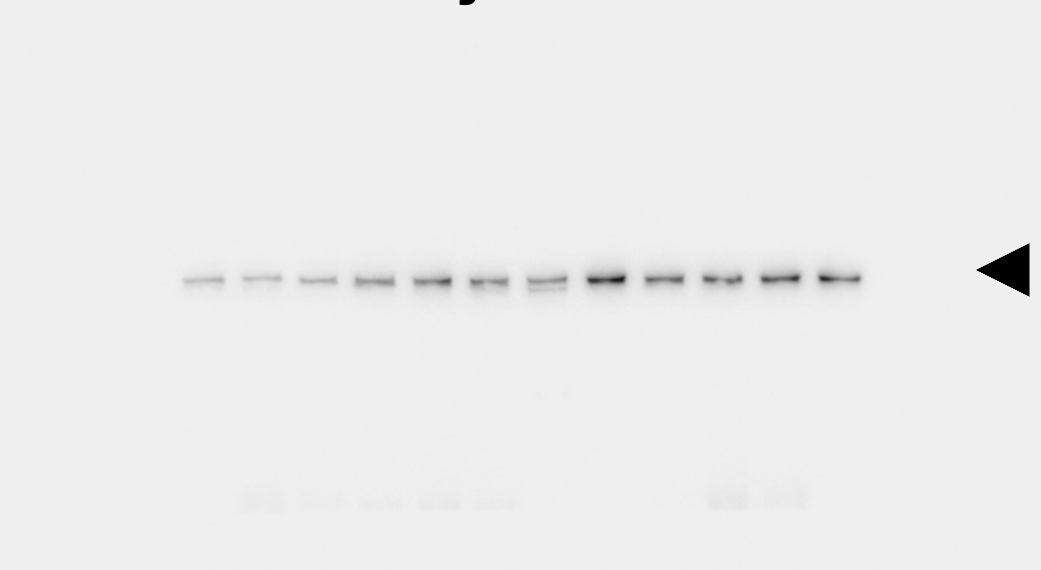

Serca1

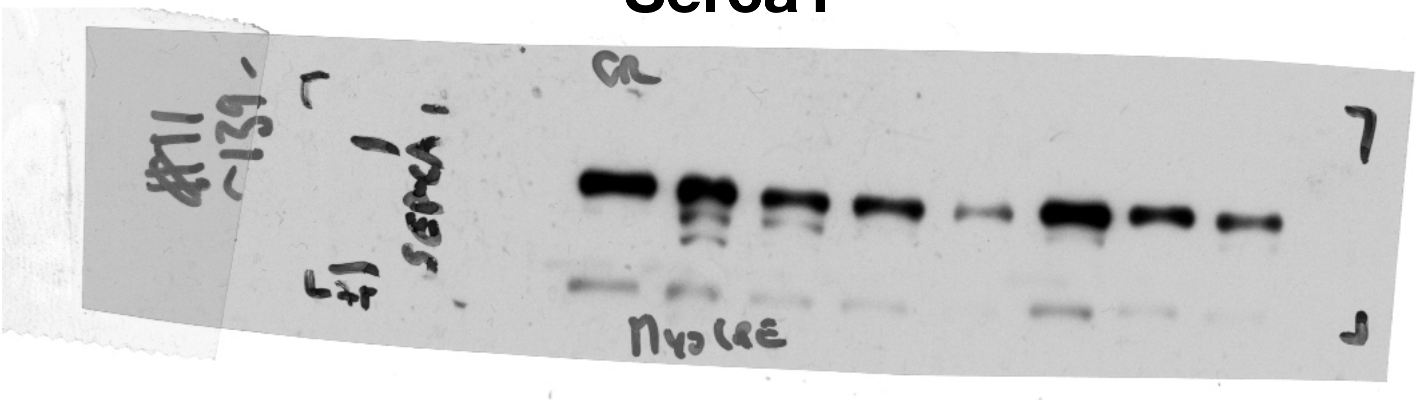

Calseq. 1

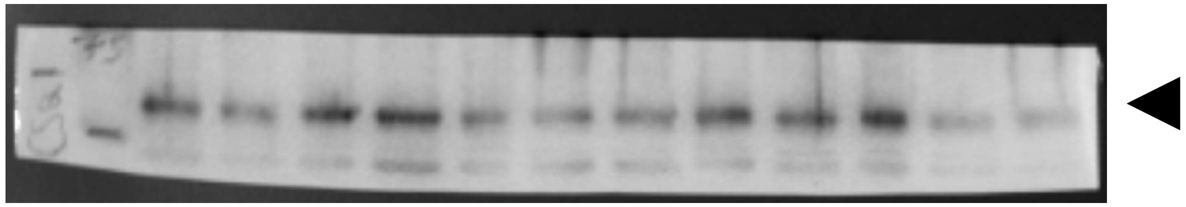

Calseq. 2

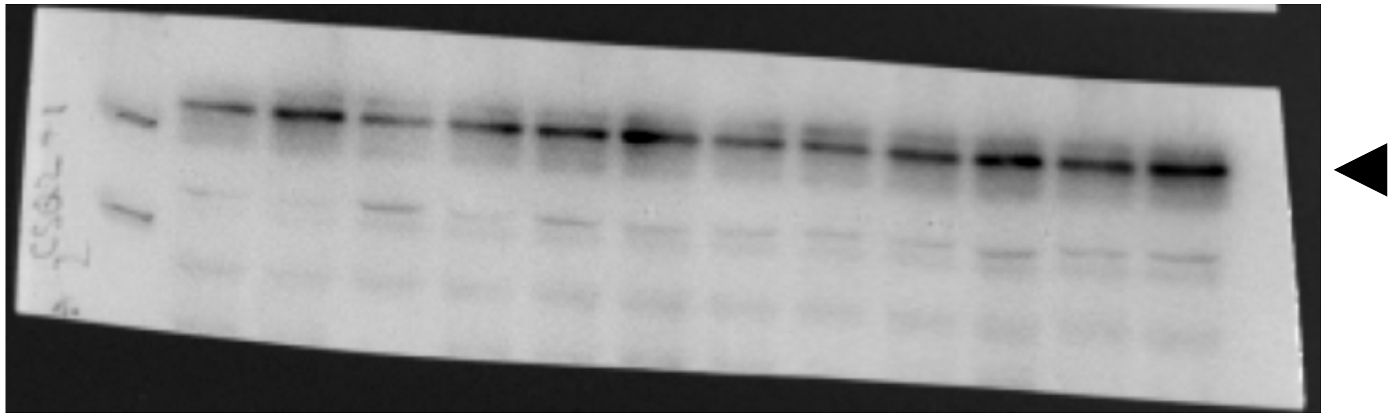

loading: lane 1=marker; lanes 2-3=controls; lanes 3-4=obscurin-ko; lanes 5-6=obsl1-ko; lanes 7-8=dKO

loading: lane 1=marker; lanes 2-3=controls; lanes 3-4=obscurin-ko; lanes 5-6=obsl1-ko; lanes 7-8=dKO
